# Targeted phasing of 2–200 kilobase DNA fragments with a short-read sequencer and a single-tube linked-read library method

**DOI:** 10.1038/s41598-024-58733-0

**Published:** 2024-04-05

**Authors:** Veronika Mikhaylova, Madison Rzepka, Tetsuya Kawamura, Yu Xia, Peter L. Chang, Shiguo Zhou, Amber Paasch, Long Pham, Naisarg Modi, Likun Yao, Adrian Perez-Agustin, Sara Pagans, T. Christian Boles, Ming Lei, Yong Wang, Ivan Garcia-Bassets, Zhoutao Chen

**Affiliations:** 1 Universal Sequencing Technology Corp., Carlsbad, CA 92011 USA; 2 Sage Science Inc., Beverly, MA 01915 USA; 3 Department of Medicine, University of California, San Diego, La Jolla, CA 92093 USA; 4 Department of Medical Sciences, School of Medicine, University of Girona, Girona, Spain; 5 Universal Sequencing Technology Corp., Canton, MA 02021 USA

**Keywords:** Haplotypes, Genetics, Molecular medicine, Genomics, Sequencing

## Abstract

In the human genome, heterozygous sites refer to genomic positions with a different allele or nucleotide variant on the maternal and paternal chromosomes. Resolving these allelic differences by chromosomal copy, also known as phasing, is achievable on a short-read sequencer when using a library preparation method that captures long-range genomic information. TELL-Seq is a library preparation that captures long-range genomic information with the aid of molecular identifiers (barcodes). The same barcode is used to tag the reads derived from the same long DNA fragment within a range of up to 200 kilobases (kb), generating linked-reads. This strategy can be used to phase an entire genome. Here, we introduce a TELL-Seq protocol developed for targeted applications, enabling the phasing of enriched loci of varying sizes, purity levels, and heterozygosity. To validate this protocol, we phased 2–200 kb loci enriched with different methods: CRISPR/Cas9-mediated excision coupled with pulse-field electrophoresis for the longest fragments, CRISPR/Cas9-mediated protection from exonuclease digestion for mid-size fragments, and long PCR for the shortest fragments. All selected loci have known clinical relevance: *BRCA1*, *BRCA2*, *MLH1*, *MSH2*, *MSH6*, *APC*, *PMS2*, *SCN5A*-*SCN10A*, and *PKI3CA*. Collectively, the analyses show that TELL-Seq can accurately phase 2–200 kb targets using a short-read sequencer.

## Introduction

Heterozygous sites occur approximately every 1–2 kilobases (kb) in the human genome, with the frequency influenced by genetic ancestry and admixture^1–6^. Phasing these sites is critical for unraveling human evolution and gaining insights into the genetic basis of complex traits and diseases^7,8^. However, accurate phasing is reliant on capturing long-range information, which is not possible with a short-read sequencing platform, such as Illumina®, unless employing a specialized library preparation method. The introduction of sequencing platforms capable of processing 20–100 kb reads (i.e., long reads), such as Pacific Biosciences® (PacBio) and Oxford Nanopore Technology® (ONT), provides confidence to phasing by allowing contiguous sequencing along multiple heterozygous sites^9^. Nonetheless, these platforms still exhibit limited genotyping fidelity and throughput when compared to short-read instruments, particularly for reads exceeding 20 kb, and remain less cost-effective^10^.

Linked-read technology offers the option to incorporate long-range information within a range of up to 200 kb into short reads, which enables phasing with the benefits of Illumina’s accuracy, throughput, and low cost^11^. Linked-read technology leverages a process of co-barcoding for reads originating from the same long DNA fragment. Thousands to millions of simultaneous DNA co-barcoding reactions enable the phasing of an entire human genome. An alternative co-barcoding method, known as synthetic long reads, also allows the capturing of long-range information, but only within a genomic distance of approximately 6–10 kb^12–14^.

There are three major linked-read methods based on the compartmentalization strategy of co-barcoding reactions: droplet/microfluidics-based methods^15–20^, microwell/plate-based methods^11,21,22^, and microbead/single tube-based methods^23,24^. TELL-Seq™ (Transposase Enzyme Linked Long-read Sequencing) is a microbead-based linked-read method that leverages a viscous solution to effectively ‘compartmentalize’ millions of co-barcoding reactions in a single tube^24^. The use of TELL-Seq contributes to the robustness and versatility of short-read platforms in capturing long-range genomic information. Nonetheless, while TELL-Seq was developed to phase entire human genome and assemble metagenomes, it remains unclear whether the technology will be effective for phasing single loci in targeted applications.

When phasing a limited number of DNA targets with TELL-Seq, the primary concern is target collisions—specifically, the co-binding of two or more individual target molecules on the same microbead, because collisions of maternal and paternal copies of the same locus disrupt the phasing process. For whole genomes, a certain number of collisions is deemed acceptable and improve cost-efficiency given the unlikely event that a maternal and paternal copy of the same locus will collide on the same microbead. However, with targets, the implicit lack of sequence diversity in a sample increases the likelihood of collisions between maternal and paternal copies of the same locus.

The presence of off-target DNA accompanying a target is expected to add sequence diversity and exert a permissive influence on collision acceptance, critical in defining the proper conditions for high-quality targeted phasing. There are at least three classes of pre-enrichment methods, each contributing varying levels of off-target DNA: probe-based by hybridization^25,26^; CRISPR-based by RNA-guided Cas9 nuclease-mediated excision^27^; and long-range PCR^18^. However, besides target purity, we suspect that other factors may impact the process of phasing, such as target size and heterozygosity density. Here, we report and validate TELL-Seq conditions generating high-quality phased data for diverse target sizes, off-target background amounts, and heterozygosity.

## Results

### Adapting whole-genome sequencing (WGS) TELL-Seq to targeted applications

**Figure 1:**
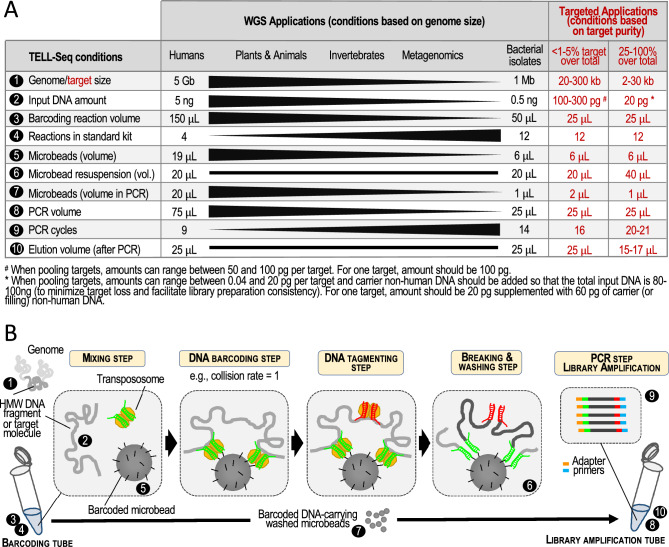
WGS and Targeted TELL-Seq protocols. (**A**) The TELL-Seq protocol was developed to assemble genomes and metagenomes, and for phasing. Although there is only one protocol, the specific amounts of starting material and TELL microbeads, as well as some aspects in the barcoding and library amplification reactions must be adjusted according to WGS applications (i.e., genomes size and sample complexity). For example, the recommended volume for the barcoding reaction is 150 μL when processing a human genome and 50 μL when processing a bacterial isolate. In this study, we propose conditions to adapt the WGS TELL-Seq protocol to the particularities of targeted applications: 1–10 (use workflow in B as reference). Based on target purity (i.e., the amount of off-target DNA or background), we anticipate two major classes of targets: largely impure targets, representing less than 1–5% of the total amount of DNA in the sample; and largely pure targets, representing 25–100% of the total amount of DNA in the sample (see Targeted Applications columns). Intermediate situations would be adjusted on a case-by-case basis, using as reference the examples provided in this study. (**B**) Briefly, ultra-low amounts of high-molecular weight (HMW) genomic DNA are mixed with transpososome and barcoded TELL microbeads. DNA (target for targeted applications) is then captured by transpososome and tagged. Transpososomes allow DNA recruitment on microbeads based on universal sequence homology. There are millions of microbeads in a TELL-Seq reaction, and each microbead provides a unique barcode to tagged DNA. A second transpososome complex allows tagging an intermediate position in the barcoded fragment and breaking and washing release transposase components. Barcoded DNA is finally released and amplified to build a library for sequencing.

We capitalized on our experience in adapting WGS TELL-Seq to the processing of genomes of different sizes to devise a series of protocol modifications that should enable high-quality targeted phasing as summarized in Fig. 1A,B. We anticipated that the key elements of an optimized TELL-Seq protocol for targeted applications would include: (i) using low amounts of input material and TELL microbeads (e.g., 100 pg or less and approximately 2 million microbeads or 6 μL in a 25 μL barcoding reaction, respectively); (ii) subsampling the amount of TELL microbeads before library amplification (e.g., using only 0.75 μL out of 6 μL for PCR); and, (iii) increasing the number of PCR cycles during library amplification (e.g., twenty-one). These adjustments aim to balance sufficient linked-read efficiency and library yield with a low risk for DNA collisions and sequencing costs. The specific volumes, amounts, and number of PCR cycles will depend on the level of target enrichment, the amount of off-target background, and the target size, as examined in this study.

### Phasing long, partially enriched targets

In general, we anticipate two major types of targets based on target size and purity: relatively large, partially enriched targets (between 15 and 200 kb with up to 95% off-target background); and relatively small, highly enriched targets (between 2 and 15 kb with less than 1–2% off-target background). To test the optimized TELL-Seq protocol with the first type of targets, we focused on seven genomic regions associated with hereditary predisposition to cancer: *BRCA1* (target length: 198.4 kb), *BRCA2* (target length: 187.5 kb), *APC* (target length: 199.6 kb), *MSH2* (target length: 201.1 kb), *MSH6* (target length: 197.5 kb), *MLH1* (target length: 201.7 kb), and *PMS2* (target length: 197.5 kb).

**Figure 2:**
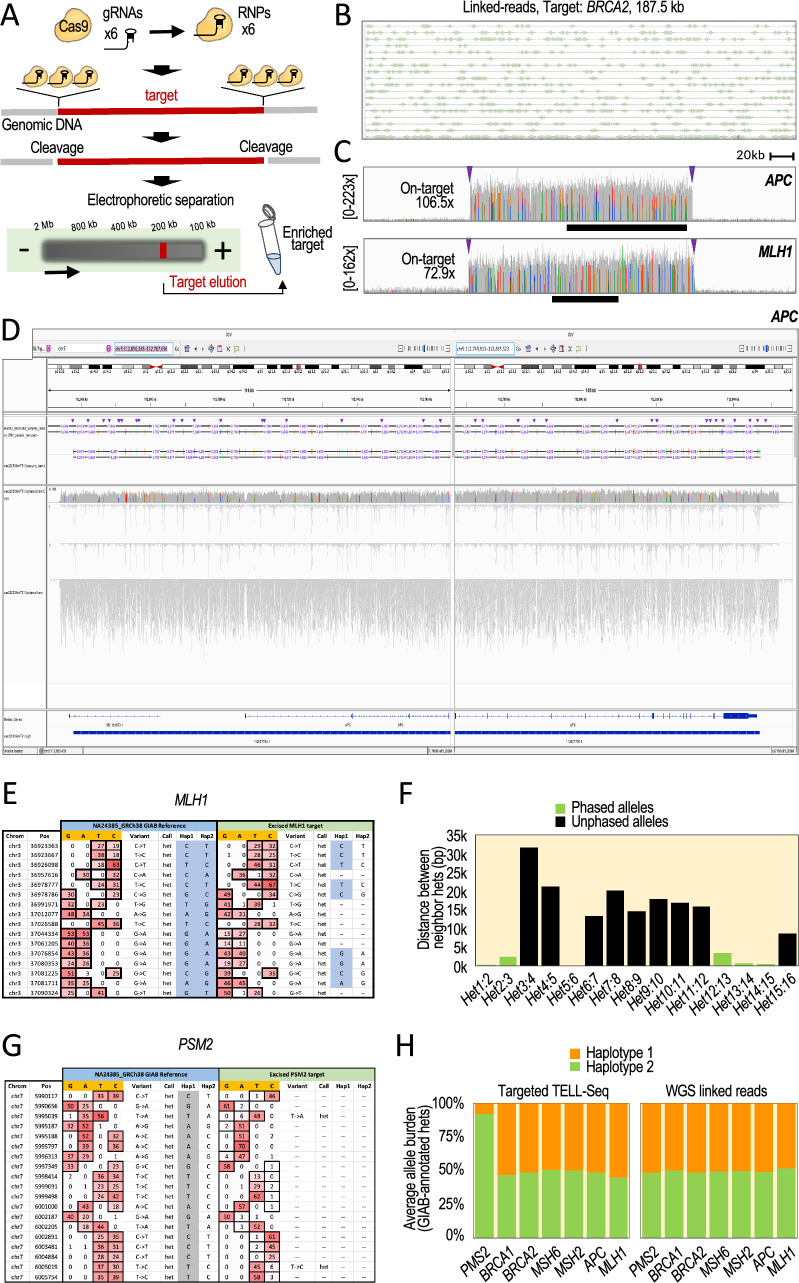
Phasing 185–205 kb HLS-CATCH-enriched targets. (**A**) Experimental workflow. See also Supplementary Fig. S1A. (**B**) A representative example of linked reads obtained with the targeted TELL-Seq protocol. Shown a 186 kb region in the *BRCA2* target. Short reads (boxes) with identical barcode are connected by lines (i.e., linked reads). (**C**) Read profiles (coverage indicated) from TELL-Seq libraries showing robust on-target recovery compared to background. Targets: 200 kb *APC* and *MLH1*. Arrowheads represent locations of a trio of gRNA binding sites on each target end. Black bars represent the *APC* and *MLH1* genes. (**D**) Joint screenshots from the Integrative Genomics Viewer (IGV) Portal showing TELL-Seq results for the *APC* target. Tracks (from top to bottom): GIAB phased haplotypes in HG002 (benchmark); phased haplotypes (numbers indicate distances between some phased sites; coverage; inferred haplotype 1; inferred haplotype 2; unphased reads; gene annotations; and inferred phase block. (**E**, **G**) Read behaviors across representative regions in the *MLH1* (**E**) and *PMS2* (**G**) targets showing correct genotyping but incomplete phasing (squared cells) and heterozygosity loss (incomplete genotyping and phasing) affecting Hap2 (squared cells), respectively. Left columns represent GIAB data (NA24385), right columns represent TELL-Seq data. Counts per positions are indicated by nucleotide G, A, T, C columns). Variants calls are also indicated (Variant columns). (**F**) Distances between the annotated neighbor heterozygous sites. Green columns represent phased sites. Black columns represent unphased sites. (**H**) Average read counts (allele burden) by target relative to the total read count. Sites selected based on GIAB annotated haplotypes.

To isolate these loci, all within the 185–205 kb range, we used the HLS-CATCH method, which can isolate targets of up to 1 Mb in size^27^. In the pipetting-free HLS-CATCH system, targets are excised from lysed cells using guide (g)RNA-directed Cas9 ribonucleoprotein (RNP) complexes, and the excised fragments are isolated using preparative pulse-field electrophoresis (Fig. 2A and Supplementary Fig. S1A)^27–30^. CATCH-isolated targets are highly enriched relative to off-target DNA (typically, 150-fold and more), but still they can represent a relatively small fraction of the total extracted DNA (e.g., 0.5–5%). We postulated that this high level of off-target background should be almost as permissive to DNA collisions as those allowed when processing a small bacterial genome (Fig. 1, table, targeted application: < 1–5% target DNA over total DNA).

We designed three gRNAs complementary to each end of every target to maximize the options of excision, minimizing also the risk of haplotype dropout due to target-specific allelic differences at the gRNA binding site (Fig. 2A). We assembled Cas9 RNP complexes in three pools: a pool with six *BRCA1*-targeting gRNAs, another pool with six *BRCA2*-targeting gRNAs, and a third pool with thirty gRNAs targeting the *APC*, *MSH2*, *MSH6*, *MLH1*, and *PMS2* loci. As source of genomic DNA, we used GM24149/HG003 and GM24385/HG002 B lymphocytes from Genome in a Bottle (GIAB), two well-characterized reference materials. We used HG003 DNA (NA24149) to isolate the *BRCA1* and *BRCA2* targets and HG002 DNA (NA24385) to isolate the other five targets.

It is important to highlight that HG002 has two sequence singularities relevant to our phasing purposes. The first singularity is a low heterozygosity density across the *MLH1* locus with a median distance between adjacent heterozygous sites of more than 13 kb, which is more than nineteen times the median distance between heterozygous sites in the other six targets (Supplementary Fig. S1B). The second singularity is the presence of a large polymorphic inversion relocating the three 3′ gRNA binding sites more than 700 bp downstream from the expected positions in the *PMS2* locus^31,32^. We confirmed this inversion using GIAB-generated WGS linked-read data (Supplementary Fig. S1C). The mixture of typical and singular targets should help us better understand the process of targeted phasing with TELL-Seq.

After recovery from the HLS-CATCH system, we mixed 100 pg of each of the *BRCA1* and *BRCA2* targets to generate a single TELL-Seq library and used 270 pg of the *APC*, *MSH2*, *MSH6*, *MLH1*, and *PMS2* co-enriched mixture to generate a second TELL-Seq library. After sequencing and duplicate removal, we obtained 10.4 million and 23.1 million reads, respectively. The mapping profiles of linked-read data were consistent with those found in conventional WGS TELL-Seq (Fig. 2B). On-target read recovery was 3.7% and 5.4% with an average mean coverage of 85.4 × and 108.5 ×, respectively. Figure 2C shows representative read density profiles for two targets.

As expected, the lowest recovery was observed for the targets with genomic singularities in HG002, 0.9% on-target recovery and 72.9 × average mean coverage for *MLH1* and 0.4% on-target recovery and 36.1 × average mean coverage for *PMS2*, compared to 1.9% and 106.4 × for *BRCA1*, 1.8% and 108.5 × for *BRCA2*, 1.3% and 106.5 × for *APC*, 1.4% and 112.9 × for *MSH2*, and 1.2% and 98.3 × for *MSH6*. We correctly phased 808 out of the 809 GIAB-annotated heterozygous sites in the five loci without singularities, which represents a 99.88% phasing accuracy: 308 out of 308 for *BRCA1*; 71 out of 71 for *BRCA2*; 110 out of 110 for *APC*; 195 out of 195 for *MSH2*; and 124 out of 125 for *MSH6*. The only phasing inconsistency with GIAB data was a flip error along the *MSH6* locus—a swap of maternal and paternal alleles. Importantly, we were also able to infer a single block of contiguous phased positions in all targets (for example, Fig. 2D, see a single blue line at the bottom; the apparent break results from joining two screen snapshots). Furthermore, we were able to de novo phase twenty-four GIAB-annotated-but-unphased heterozygous sites in our tests: 8 sites in *APC*, 13 sites in *MSH2*, and 3 sites in *MSH6*. However, we missed four GIAB-annotated heterozygous sites due to an insufficient read recovery underlying these sites in the *APC* and *MSH6* targets, which could be considered as failed genotyping.

For the singular *MLH1* region in HG002, genotyping was 100% accurate (16 out of 16 correctly recalled heterozygous sites), but phasing was largely incomplete with only 9 correctly phased positions out of 16 (56%, Fig. 2E, Hap1 and Hap2 columns). Moreover, we could not infer a continuous phase block for this target. We attributed this low performance to an insufficiency of linked reads among heterozygous sites, which were more than 10 kb apart (Fig. 2F), combined with a relatively low read coverage compared to the other targets.

For *PMS2*, the second target with a singularity in HG002, all GIAB-annotated heterozygous sites were incorrectly recalled as homozygous in our data, which is consistent with a full haplotype dropout generated by the large inversion (Fig. 2G). In agreement, we observed a high coverage imbalance between the two sets of GIAB-annotated alleles with an average of 91.98% and 8.02% relative abundance, compared to 48.52% and 51.48% in HG002 GIAB WGS data (Fig. 2H). Interestingly, the alleles of the ‘lost’ haplotype were still detectable to some degree (8.02%), suggesting that there is some level of inversion mosaicism affecting primarily one of the two haplotypes (Fig. 2G, compare A, C, G, and T counts), which is also supported by a multiplicity of breakpoints inferred from WGS linked-read data (Supplementary Fig. S1C, large heatmap).

Together, these analyses show that the targeted TELL-Seq protocol is an efficient method for phasing long, partially enriched targets. However, for targets with an unusually low density of heterozygous sites, we have observed an expected low linked-read rate, which is detrimental of the phasing process. Likewise, for targets with large inversions, our analyses highlight the risk of using size-based selection as DNA pre-enrichment method. We note that some frequency of large inversion polymorphisms (e.g., 700 kb and more) have been reported in the human population^31^.

### Phasing mid-size, partially enriched targets

**Figure 3:**
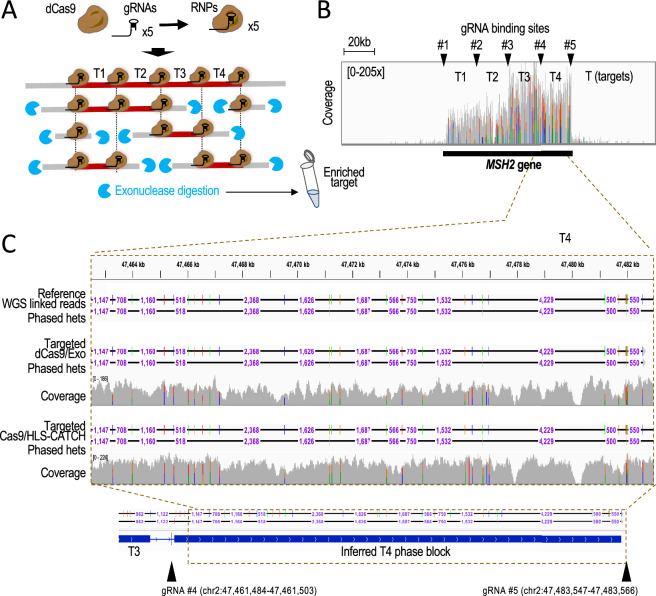
TELL-Seq-mediated phasing of four adjacent 20 kb targets enriched with dCaBagE, a modified CaBagE method that leverages dCas9 to protect end and internal sites of the target. (**A**) Experimental workflow. The entire (80 kb) or sub-targets (20, 40, or 60 kb) can be individually phased based on the quality of the input genomic DNA and the presence of free DNA ends, which serve as sites for exonuclease initiation of degradation. The protected DNA (target) is represented in dark red; unprotected DNA (non-target) is represented in grey. (**B**) Coverage across the *MSH2* locus (black bar indicates the position of the *MSH2* gene). This profile supports high on-target recovery over background for all sub-targets (T1–T4) but higher for T3–T4 than for T1–T2. Arrowheads indicate gRNA binding sites (#1–#5). (**C**) Screenshot from the IGV Portal showing phased data and coverage using targeted TELL-Seq with dCas9/exonuclease or HLS-CATCH/Cas9 as DNA pre-enrichment methods. Shown on top HG002 WGS linked-read phased data (benchmark). Phased data includes distances between phased sites (we note that distances are not shown when not allowed at the selected resolution on IGV). Bottom tracks show phased data and inferred phase blocks with targeted TELL-Seq using dCaBagE. Arrowheads indicate gRNA binding sites.

Next, we tested the targeted TELL-Seq protocol with mid-size, partially enriched targets. We used a modified version of the CaBagE protocol (Cas9-based Background Elimination)^33,34^, which leverages the stability of the Cas9/gRNA RNP complex bound to each end of a target to protect it from exonuclease digestion. Our modification consists of the use of a catalytically deactivated Cas9 form (dCas9) that enables dCas9 binding within the target—referred to as dCaBagE (Supplementary Fig. S2A). In the reaction, dCas9 binds to the target DNA, thereby shielding specific genomic segments from degradation by exonucleases (Supplementary Fig. S2A,B). This strategy can be applied to protect mid-size target segments in DNA extracted using pipetting-based procedures, in which high-molecular weight (HMW) genomic DNA will be invariably fragmented even for HMW DNA methods, generating fragments not longer than a few kb. As target, we selected a 80 kb region in the *MSH2* locus, and designed gRNAs to guide dCas9 to the flanks of this region and along internal intervals of 20 kb, which could protect subfragments hereafter referred to as T1-T4 (Fig. 3A and Supplementary Fig. S2B)^35^. Such gRNA strategy based on five RNP binding sites and complexes (#1–#5) should enable the recovery of 20 kb, 40 kb, 60 kb, and 80 kb fragments if fragments of all these sizes were preserved during genomic DNA extraction^36^. The most frequent expected target size would be 20–40 kb when working with a genomic DNA prep showing average fragment sizes in the 30–50 kb range (as in our case).

After RNP assembly, we incubated the pool of five RNP complexes with 1 μg of HG002 HMW genomic DNA followed by exonuclease digestion. We then applied the targeted TELL-Seq protocol used for HLS-CATCH-enriched targets but with approximately half of the amount of starting DNA material. After sequencing and duplicate removal, we obtained 6.3 million reads with 0.8% on-target recovery and 68.7 × average coverage depth.

Overall, T3 and T4 were more efficiently recovered than T1 and T2 (0.3%, 0.3%, 0.1%, and 0.1% on-target recovery and 104.5 ×, 85.6 ×, 40.5 ×, and 44.9 × average mean coverage, respectively), suggesting that RNPs #3–5 provided better protection than RNPs #1–2 (Fig. 3B). In addition, T3 and T4 showed a 100% recall and phasing accuracies: 27 out of 27 and 32 out of 32, respectively (Fig. 3C, T4). Also, T3 and T4 could be both inferred as a single block, suggesting that ~ 40 kb and larger fragments were rare in the sample of genomic DNA (Fig. 3C, bottom). For the underperforming T2, three GIAB-annotated heterozygous sites (out of 33) were missed by our method (Supplementary Fig. S2C). The rest of T2 heterozygous sites (30) were recalled and correctly phased except for one site that remained unphased (Supplementary Fig. S2D), thus representing a ~ 96.7% phasing accuracy of recalled sites. For the underperforming T1, phasing was more problematic. Despite that all heterozygous sites (9 out of 9) were recalled, two sites could not be phased, leading to a ~ 77.8% phasing accuracy (Supplementary Fig. S2E,F). Additionally, we inferred a discontinuous phase block for T1 (Supplementary Fig. S2E,F, bottom track). As in the case of the *MLH1* locus in HG002, we attributed the problems with T1 to a distinctively low heterozygosity (9 heterozygous sites in T1 compared to 27, 32, and 30 in T2–T4). Nonetheless, our analyses reveal no flip or switch phasing errors. Overall, these results demonstrate that TELL-Seq is also capable of phasing partially enriched, mid-size targets, although a target with distinctively low heterozygosity might be challenging.

### Minimizing DNA collisions with highly pure targets

Next, we sought to validate the targeted TELL-Seq protocol for relatively short but highly pure targets (e.g., PCR amplicons). Phasing amplicons will require further optimization of the protocol. For instance, whole-genome TELL-Seq tolerates an average of 6–8 collisions on the same microbead due to the extremely low possibility that maternal and paternal copies of the same locus collude on the same microbead^24^. In contrast, an average of 6–8 collisions on the same microbead would be detrimental when processing a single amplicon due to the almost certain possibility that maternal and paternal copies of the amplified locus will collude on the same microbead. Additionally, amplicons will require a much lower barcode diversity for phasing than whole genomes or partially enriched targets.

**Figure 4:**
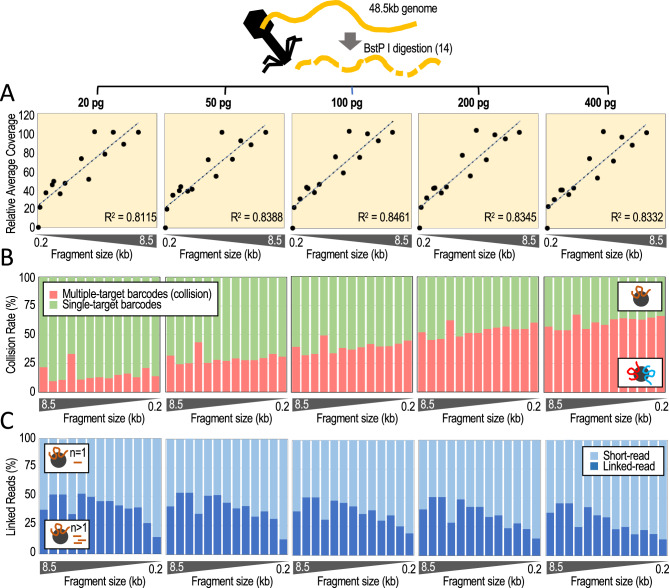
Determining collision rates and linked-read efficiencies using BstP I-digested lambda phage genomic DNA. (**A**) Relative coverage (average across every fragment) relative to fragment size. On top indicated input amounts of digested fragments. Y-axis represents the average coverage relative to the average coverage with the 8.5 kb fragment, X-axis represents fragment size (each datapoint is one fragment). (**B**) DNA collision rates (%) relative to fragment size by input amounts. (**C**) Percentage of linked reads relative to the total number of short reads by fragment size and input amounts. Analysis limited to microbeads with only one associated fragment (single-barcode fragments or collision-free cases).

To determine conditions less likely to generate collisions, we generated five TELL-Seq libraries using 20, 50, 100, 200, and 400 pg of the BstP I-digested lambda phage genome. BstP I digestion splits the 48.5 kb lambda phage genome into fourteen non-overlapping fragments, which mimics a pool of 0.1–8.5 kb targets with identical number of molecules in every case (117, 224, 702, 1264, 1371, 1929, 2323, 3675, 4324, 4822, 5687, 6369, 7242, and 8453 bp). This diversity should allow us to quantify collisions with the only caveat that coincidence of two molecules of the same fragment will be missed. After sequencing the libraries, we recovered all but the 117 bp fragment (Supplementary Fig. S3A). On-target recovery correlated with fragment size (R^2^ = 0.8111–0.8461; Fig. 4A), although the fragment ends of every target showed distinctively lower coverage (Supplementary Fig. S3B). The highest fraction of collision-free barcoding events corresponded to the 20 pg input (84.8 ± 6.5%) and the lowest fraction corresponded to the 400 pg input (38.7 ± 4.8%), gradually decreasing with increasing DNA amounts: 50 pg, 70.7 ± 4.9; 100 pg, 61.0 ± 4.7; and 200 pg, 46.5 ± 5.1 (Fig. 4B). Within the fraction of estimated collision-free barcoding events, we also calculated linked-read efficiencies (i.e., linked reads over total short reads). Linked-read efficiency was the highest with the 20 pg input (40.9 ± 10.9%) and the lowest with the 400 pg input (29.4 ± 11.3%), but relatively uniform between both amounts: 50 pg, 40.6 ± 11.2%; 100 pg, 37.7 ± 9.6%; and 200 pg, 35.4 ± 11.4% (Fig. 4C). As expected, linked-read efficiencies increased with coverage (Supplementary Fig. S3C), but showed no strong dependency on fragment size when normalized by coverage for 0.7 kb and larger fragments (Supplementary Fig. S3D).

### Phasing amplicons

We have estimated that 20 pg target inputs allows ~ 85% collision-free DNA co-barcoding events (Fig. 4B). The caveat is that, in our experience, inputs of 20 pg and lower can generate inconsistent library yields. We, therefore, reasoned that consistency in library yields could be achieved by adding 60–80 pg of non-human fragmented DNA, hereafter referred to as ‘filling’ DNA (e.g., *Escherichia coli* or BstP I-digested lambda phage genomic DNA). To test whether 20 pg inputs supplemented with 60–80 pg of filling DNA are suitable conditions for efficient phasing, we aimed to generate a series of long PCR products to use as input material.

Long-range PCR (3–30 kb) can be notoriously challenging, especially when amplicons are used for the purpose of phasing. First, long-range PCR often requires substantial optimization^37,38^. Second, PCR conditions must be established that reduce the risk of haplotype dropouts and chimeras. Haplotype dropouts can be generated when either the maternal or paternal copy of the target is inefficiently amplified^39^. Chimeras are crossovers of maternal and paternal haplotypes and can occur when an incomplete PCR product acts as a primer on the wrong amplicon copy during PCR amplification, leading to a switch error (or an artificial haplotype)^40^. In part, the risk of haplotype dropouts and chimeras can be reduced by using long extension times and avoiding over-amplification. In this context, it is advantageous that TELL-Seq requires only picogram amounts of input DNA, which allows minimizing the number of PCR cycles.

With these precautions, we amplified a 13 kb region in the *SCN5A*–*SCN10A* locus containing seven polymorphic sites near gene-regulatory elements associated with a high risk for sudden cardiac arrest^41–43^. These seven sites are spaced 288, 4299, 23, 69, 5560, and 2505 bp apart (from 5′ to 3′)^43^. As a second target, we selected the 3.4 kb region spanning the exons of the *PIK3CA* gene. *PIK3CA* is the second most frequently mutated gene across all cancer types, and it is mutated at least twice (i.e., double mutations) in 8–13% of clinical cases^44,45^. Phasing double *PIK3CA* mutations can help to predict oncogenicity and sensitivity to treatment, such as when the E545K and L866F mutations coincide in the same chromosomal copy (in cis)^45^. These mutations can also be in a different chromosomal copy (in trans) relative to a nearby allelic variant, I391M (rs2230461)^45^. The distances between these three sites are 459 and 964 bp in cDNA.

**Figure 5:**
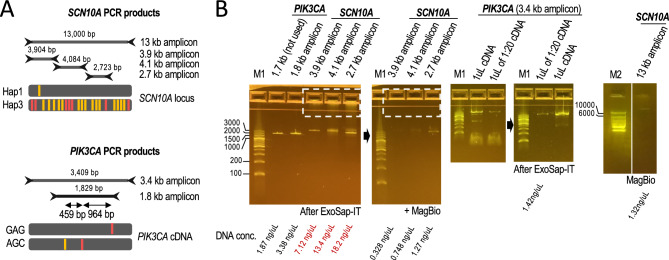
Amplicons used in this study. (**A**) *SCN10A* and *PIK3CA* amplicon sets phased in this study (13, 3.9, 4.1, and 2.7 kb from genomic DNA and 3.4 and 1.8 kb from cDNA, respectively). HG001 carries previously annotated Hap1 and Hap3 haplotypes. Red bars indicate the position of 7 clinically relevant heterozygous sites; orange bars indicate the position of 15 additional heterozygous sites. Alleles are colored if represent the alternate allele, i.e., different from the human reference. HCC202 cells carry two clinically relevant alleles (in red) and one non-clinically relevant variant (in orange). (**B**) 2% E-gels and 2% agarose (for 13 kb fragment) with PCR products as indicated. Non-specific DNA and primers were removed as indicated (e.g., compare DNA traces before and after MagBio cleanup, dashed squares; see Methods for technical details). We selected minimally amplified PCR products (see 3.4 kb *PIK3CA* 1:20 diluted cDNA as an example). DNA concentrations are indicated at the bottom.

For our phasing analyses, we amplified the 13 kb *SCN10A* and 3.4 kb *PIK3CA* fragments as well as shorter products within the same regions to examine the reproducibility of the phasing data: 3.9, 4.1, and 2.7 kb for *SCN10A* (from 5′ to 3′); and 1.8 kb for *PIK3CA* (Fig. 5A). For *SCN10A* targets, we used the GIAB reference HG001 as a source of genomic DNA (NA12878). Analysis of WGS linked-read data^24,32^ reveals that HG001 is a carrier of so-called Hap1 and Hap3 haplotypes in the *SCN5A*-*SCN10A* locus (TACCATT and CGGGGGC)^43^. Further analysis of the same data reveals that HG001 carries another subset of fifteen heterozygous sites, some of which have also been recently associated with heart arrhythmia^41^. The twenty-two heterozygous sites are split by major and minor allele frequencies, which builds upon the previously proposed model that the 13 kb Hap1/Hap3 genotype represents fully separated all-major/all-minor alleles^43^. On the other hand, to generate *PKI3CA* amplicons, we used cDNA generated from human breast cancer HCC202 cells. HCC202 cells carry the GAG and AGC haplotypes underlying the three heterozygous sites under investigation (E545K, L866F, and I391M)^45^. All amplicons appeared as weak, mainly single PCR products on E-gels (before cleanup), suggesting no over-amplification (Fig. 5B), and were generated without major apparent haplotype biases (Supplementary Fig. S4).

**Figure 6:**
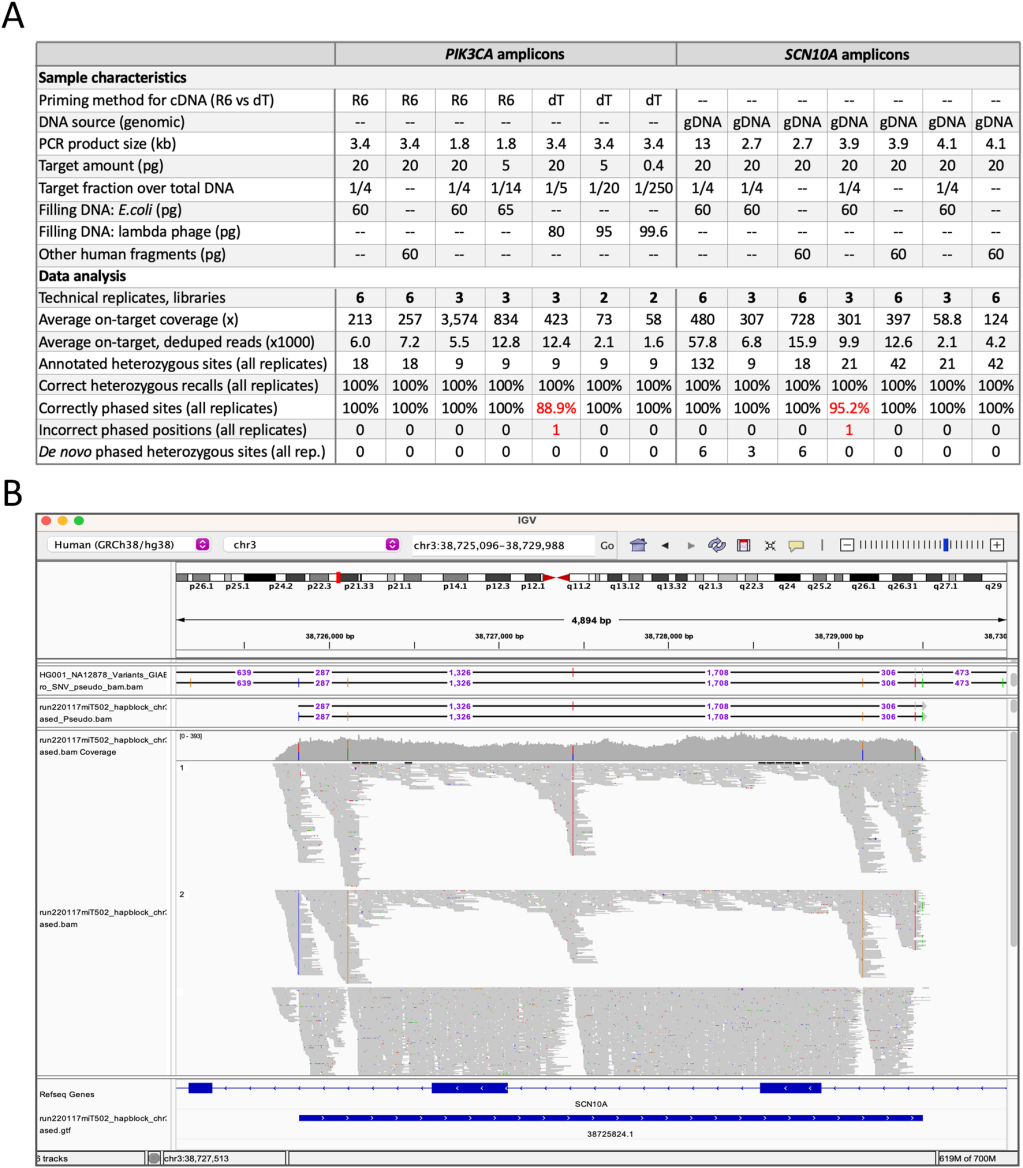
Phasing *SCN10A* and *PIK3CA* amplicons (sizes: 1.8–13 kb). (**A**) Summary table of genotyping and phasing results using *SCN10A* and *PIK3CA* amplicons as input DNA; PCR from NA12878 genomic DNA (n = 33 tests) or breast cancer HCC202 cDNA (n = 25 tests). In red, highlighted the phasing errors. R6 (random hexamers) and dT (oligo-dT) for cDNA priming, as indicated. (**B**) Screenshot showing the phasing of three technical replicates on the IGV Portal. Fragments corresponds to 4.1 kb *SCN10A* amplicon. The top track shows the phasing of one 4.1 kb replicate in phased.pseudo.bam format. Heterozygous sites are separated by numbers that represent distances between neighboring sites. The third track show coverage, inferred haplotype 1 reads, inferred haplotype 2 reads, and unphased reads. The last tracks show gene annotations and an inferred phase block (blue bar).

For a comprehensive analysis, we generated forty TELL-Seq libraries to assess different amplicon amounts, target sizes, and cDNA priming methods, as well as to acquire replicates (between 2 and 6). Six of these libraries were generated with a pool of amplicons (3.4 kb *PIK3CA* and 2.7, 3.9, and 4.1 kb *SCN10A*). The total input amounts varied from 5 to 20 pg with one case using as low as 0.4 pg (Fig. 6A, target amount), and were supplemented with filling DNA when processing single targets (either *E. coli* genomic DNA or BstP I-digested lambda phage genomic DNA), or without filling DNA when processing a pool. For pools, we used equal amounts for each target (20 pg). The rationale for using equal amounts for each target in a pool (rather than equal number of molecules) is that the expected lower coverage for small fragments should be compensated by the higher molecule number compared to large fragments, as suggested by our analyses with the digested lambda phage genome (Fig. 4). For cDNA-based products, we tested reverse transcriptase primed by random hexamers and oligo-dT. Finally, we mapped *SCN10A* reads to genomic DNA and *PIK3CA* reads to the target *PIK3CA* cDNA with the allele frequency (AF) cutoff set to 0.1 (see Methods). An integrated analysis of all these experiments revealed that the average on-target coverage ranged between 58.8 × and 3574 × with most cases falling between 200 × and 800 × (Fig. 6A, average on-target coverage).

Out of the 366 annotated heterozygous sites—collecting the numbers from the fifty-eight replicates—we correctly phased 364, while the remaining two sites represented flip errors. Overall, we achieved 100% genotyping and 99.45% phasing accuracies (Fig. 6A,B). Importantly, the two flip errors were not reproduced in replicate samples. Additionally, we genotyped and phased a heterozygous site not previously annotated by GIAB, but that it was consistent across fifteen libraries (Fig. 6A, de novo phasing). Together, these results indicate that targeted TELL-Seq can achieve highly accurate phasing of amplicons when supported by replicates.

### Benchmarking TELL-Seq with long-reads

**Figure 7:**
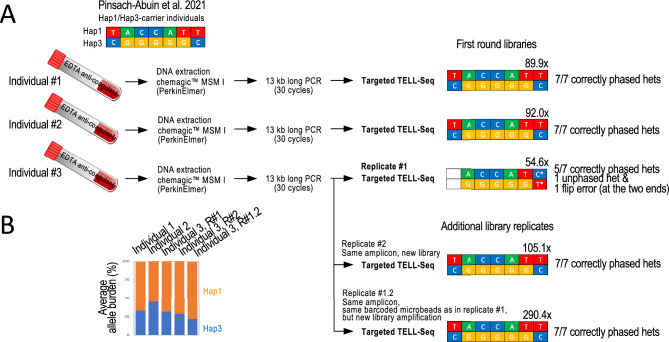
Targeted TELL-Seq with three 13 kb amplicons generated from peripheral blood-extracted genomic DNA from three known Hap1/Hap3 *SCN10A* carrier individuals. (**A**) Previous work identified the three Hap1/Hap3 carrier individuals from whom 13 kb amplicons were generated and now have been processed in this study. We confirmed the Hap1/Hap3 genotype in all three individuals with targeted TELL-Seq. However, this conclusion needed the three replicates for Individual #3. In Replicate #1, we observed an unphased and a flip error. We note that this library provided the lowest coverage, 54.6x (Individual #3). We generated a new library from the same amplicon (Replicate #2) and re-do the library amplification with leftover barcoded microbeads from Replicate #1 (Replicate #1.2). In both cases, we validated the expected Hap1/Hap3 genotype. We note that the sequencing depth was also higher for both replicates, 105.1 × and 290.4 ×, respectively. Coverage indicated (x) on top of each genotype and number of correctly re-called and phased heterozygous positions in Hap1 and Hap3 are also indicated. (**B**) Graph shows average allele burden for all Hap1/Hap3 sites by library. In all cases, Hap1 was amplified slightly more efficiently than Hap3, although it had no effect on genotyping and phasing.

Next, we benchmarked the targeted TELL-Seq protocol with previously generated long-read results of 13 kb *SCN10A* products amplified from peripheral blood-derived genomic DNA^43^. From this previous study, we selected three Hap1/Hap3 carriers (referred here to as individuals #1–3)^43^, and obtained the original amplicons. Our results agreed with the previously reported long-read-based (ONT) genotypes in individuals #1 and #2, recalling and correctly phasing the seven clinically relevant heterozygous positions in each sample (Fig. 7A, Ind.#1 and #2). For individual #3, the same seven sites were also recalled as heterozygous in our data, although the most 5’ site remained as unphased and the most 3’ site was phased inconsistently with prior data (Fig. 7A, Ind.#3, Replicate #1). Since coverage was lower for individual #3 than for individuals #1–2 (54.6 × compared to 89.9 × and 92.0 ×, respectively), we suspected that our call for the most 3’ site in individual #3 was erroneous. In support, we generated a second TELL-Seq library from the same amplicon (Ind. #3, Replicate #2) and reamplified the first TELL-Seq library from the remaining target-bound microbeads (Ind. #3, Replicate #1.2). These two new tests were based on higher coverage (105.1 × and 290.4 ×) and agreed with a Hap1/Hap3 genotype (Fig. 7A, Ind.#3, Replicate #2 and #1.2). We also noticed that linked-read coverage at the two haplotype ends in the first replicate from individual #3 had been the lowest of all tests, again connecting phasing problems to low coverage (Supplementary Fig. S5A, orange columns). For reference, the published ONT data was based on at least 600 × coverage for reads containing all seven heterozygous sites (Supplementary Fig. S5B)^43^.

On a different note, we observed a slight haplotype bias (Hap1 > Hap3) with blood-based targets that we did not observe when amplifying the 13 kb fragment from cell culture-derived HG001 DNA (compare allele burden plots between Supplementary Fig. S4 and Fig. 7B). However, this haplotype bias did not apparently have a negative effect on the process of phasing.

### Minimum sequencing depth for accurate phasing

Throughout this study, we have repeatedly observed the importance of sufficient sequencing coverage for the process of phasing. To examine in more detail how sequencing coverage (as a proxy for linked-read capture) affects genotyping and phasing accuracy with the targeted TELL-Seq protocol, we subsampled nine of the sequencing outputs generated from the libraries using amplicons and repeated the phasing analyses. We subsampled the sequencing outputs at 50%, 25%, 12.5%, 6.25%, 3.125%, 1.56%, and 0.78% of the original dataset. After duplicate removal, we segregated the data into four groups based on phasing quality. Group 1 integrates results without genotyping or phasing errors and a single phase block; group 2 integrates results without genotyping errors and a single phase block but with one flip or switch error; group 3 integrates results with incorrect genotyping, discontinuous phase blocks, and flip/switch errors; and group 4 integrates results with mostly unphased heterozygous sites (Supplementary Fig. S6). These data separation revealed that lowering the coverage below 180 × for the 13 kb fragment and 150 × for the 2.7–4.1 kb fragments led to phasing errors, and that further lowering the coverage added genotyping errors (Supplementary Fig. S6, compare group 1 and groups 2–4). Still, we note that results based on 50–60 × coverage often show no phasing errors (see cases in Fig. 6A). These subsampling tests reinforce the importance of sufficient coverage to reduce the risk of phasing errors.

## Discussion

For many applications, long-range information is essential for interpreting genomic data^17,46–53^. TELL-Seq is a single-tube, linked-read library preparation method that efficiently introduces long-range-information into short reads^24^. The original TELL-Seq protocol, developed for whole genomes and metagenomes, is permissive of DNA collisions (on average, 6–8 fragments colluding on the same microbead). However, when phasing a single locus or a few loci, TELL-Seq must minimize DNA collisions due to the low fragment diversity (complexity) of the sample. In this study, we report and validate modifications in the original TELL-Seq protocol aimed for targeted applications. This modified protocol has a version for impure targets (< 1–2% target DNA over total DNA) and a version for pure targets (> 90–95% target DNA over total DNA). Both versions can be used with single targets or target panels with only minor procedural variations. Pooling targets (i.e., panels) enables higher throughput, but it is critical that the target sequences must not overlap with each other (i.e., represent different loci). We have benchmarked the targeted TELL-Seq protocol with WGS linked-read and long-read data, demonstrating high-quality genotyping and phasing accuracies. The most problematic cases are characterized by abnormally low heterozygosity, with heterozygous positions separated by 10 kb or larger distances. These cases will require much higher coverage than typical targets (the specific amount of coverage will have to be determined empirically on a case-by-case basis).

We show that phasing quality depends on heterozygosity density and target coverage. To determine whether target coverage is sufficient to ensure high quality phasing, we suggest paying attention to the presence of unphased heterozygous positions and discontinuous phase blocks. When not all heterozygous sites have been phased or belong to the same phase block, it is a good indicator that deeper sequencing would be required to increase the quality of the phasing. We also strongly recommend generating replicates because erroneous phasing is non-reproducible. With amplicons in particular, the risk of phasing errors can also be reduced by avoiding over-amplification, and generating replicates with different primer sets. In any case, multi-band PCR products should be avoided unless there is confidence that the extra products do not represent shorter versions of the target.

Regarding input amounts, we have established that high-quality phasing data can be obtained with no more than 100 pg of an impure target and 20 pg of a pure target. When working with less than 80 pg inputs, we recommend supplementing with filling DNA for yield consistency and the highest recovery. The lowest target input amount tested is 0.4 pg (supplemented with 99 pg of filling DNA), suggesting that, at least in principle, hundreds of non-overlapping targets (e.g., 250) could be pooled in a single TELL-Seq library preparation, especially valuable for Cas9-based DNA pre-enrichment methods using hundreds of gRNAs. Pooling has the advantage of reducing library preparation costs and time. When using filling DNA, it should be either bacterial genomic DNA or digested lambda phage DNA. We used BstP I-digested phage DNA, but other options could also be used if are based on at least 10–14 fragments, regardless of the specific restriction enzyme/s used.

Regarding the DNA-pre-enrichment method, our data confirm the value of using a gentle, pipetting-free system, such as HLS-CATCH, and isolating targets in pools. The limitation of using a system that relies on size for target isolation is that unexpected genomic rearrangements may alter the size properties of the target, causing haplotype dropouts. Nonetheless, many genomic rearrangements should not have a major effect on DNA motility in pulse-field electrophoresis since it is a low-resolution DNA separation method. We also note that HLS-CATCH and dCaBagE recover a significant amount of background in addition to the target, leading to substantially low on-target read fractions; still, these methods generate high target enrichment over background, although likely dCaBagE generates more background than CaBagE, the original method.

Finally, a note about the IGV tracks showing phased TELL-Seq data throughout this study (e.g., Figs. 2D, 3C, and 6B). These tracks were generated using a new computational tool that we have recently developed that allows convenient browsing of phased variants as part of haplotypes and phase blocks and enables to examine the distance between heterozygous sites and match data with gene annotations and read coverage on the IGV Portal. This tool is now available through the Tell-Sort pipeline for TELL-Seq analysis (see Methods).

In conclusion, TELL-Seq enables working with a short-read sequencer to phase targets of different purity levels in the 2–200 kb range. It will remain to be determined whether the same performance reported here for the targeted TELL-Seq protocol can be observed for other targets and DNA pre-enrichment methods or conditions not tested in this study. We also encourage testing conditions with fewer microbeads (e.g., 3 μL or less) to improve cost-effectiveness in large scale experiments.

## Materials and methods

### Human cell sources

Human lymphoblastoid GM24149, GM27730, and GM12878 cells were purchased from Coriell Cell Repositories® (Cat. # GM24149, # GM27730, and #GM12878). Human epithelial breast cancer HCC202 cells were purchased from ATCC® (Cat. #CRL-2316).

### Peripheral blood-derived amplicons from human donors

Amplicons from three unrelated individuals were previously generated from peripheral blood-derived genomic DNA^43^. Briefly, blood was collected in EDTA anti-coagulant BD Vacutainer tubes and genomic DNA was extracted using the Chemagic MSM I Instrument (PerkinElmer) following the manufacturer’s recommendations and stored at − 20 °C before PCR^43^. The samples were obtained according to the Declaration of Helsinki Principles and complying with the European and National Code of Practice with consents approved by the Clinical Research Ethics Committee of the Dr. Josep Trueta Hospital (#2012.097).

### Genomic HMW DNA extraction and target isolation with the HLS-CATCH system

For HMW genomic DNA extraction and target isolation with the HLS-CATCH system (Sage Sciences), we followed instructions according to the manufacturer’s recommendations^27^. We used one million cells for each isolation. We performed three isolations: *BRCA1* and *BRCA2* separately and *APC*, *MLH1*, *MSH2*, *MSH6*, *PSM2* collectively using GM24149/HG003 cells for *BRCA1* and *BRCA2* and GM27730/HG002 cells for the rest of the targets. Cas9/gRNA RNP complexes were assembled with 2 μM Cas9 and the pool of gRNAs (see table below). After 4 min/80 V electrophoretic injection, each targeted fragment was size separated and eluted by pulse-field electrophoresis. This approach yielded 220,000–400,000 copies per targeted locus with an enrichment of 200–400-fold over genomic DNA (measured by quantitative PCR). The isolated DNA fractions ranged between 3 and 7 ng.

### HMW genomic DNA extraction with a modified salting-out method

For GM12878/HG001/NA12878 and GM27730/HG002/NA24385, HMW genomic DNA was extracted using a salting out method previously reported^54^ with some modifications. Five million cells were harvested at 315×*g* for 7 min in a 15 mL centrifuge tube (Beckman Coulter Allegra X-14R) and stored at − 80 °C. The cell pellet was resuspended in 3 mL of nuclei lysis buffer (10 mM Tris–HCl, 400 mM NaCl, and 2 mM EDTA, pH 8.0) by gentle inversion (20 times). For cell lysis and protein digestion, 0.2 mL of 10% SDS and 0.5 mL of Proteinase K solution (1 mg/mL Proteinase K, 2 mM EDTA, pH 8.0) were added to resuspended cells and mixed by gentle inversion (5 times), then incubated with rotation at 37 °C overnight (12–18 h). For DNA extraction, 1.2 mL of 5 M NaCl were first added and mixed for 15 s by inversion, cellular/protein debris were then collected by centrifugation at 1000×*g* for 15 min at 4 °C (Beckman Coulter Allegra X-14R), and the supernatant (DNA) was finally transferred slowly to a new 15 mL tube using a serological pipette. For DNA precipitation, 8 mL of 100% ethanol were added (200 proof [absolute] for molecular biology) and mixed by inversion (at least 10 times). A precipitate of long DNA strands should be clearly visible during this step. The DNA solution was pelleted by centrifugation at 6000×*g* for 5 min at 4 °C (Eppendorf Centrifuge 5425R). After removing the supernatant, DNA pellets were left to dry for 5 min at room temperature and resuspended in 300 μL of 0.1 × TE buffer (10 mM Tris–HCl pH 8.0, 0.1 mM EDTA). Following an overnight incubation at room temperature and a 2-day incubation at 4 °C, the DNA concentration was determined to be 50 ng/μL by the Qubit High-sensitivity dsDNA Assay (Thermo Fisher Scientific). HMW genomic DNA was stored at 4 °C for up to 2 weeks or at − 20 °C for up to 6 months. We note that the main difference between the method used here and previously published^54^ is in the DNA extraction step, which added pipetting and centrifugation steps after ethanol addition. If ultra-long HMW DNA is required, we suggest following the original protocol, which avoids mechanical DNA shearing by physically transferring the precipitated DNA strands with a plastic spatula or pipette directly into 200 μL of 0.1 × TE buffer, yet this option has the risk of losing DNA.

### Target excision and isolation with a modified CaBagE method

We modified the CaBagE method^33,34^ and designed a tiling strategy of spacing gRNAs as previously shown^36^. Five gRNAs were designed to isolate four ~ 20 kb adjacent targets (T1-T4) from the human *MSH2* locus. We designed gRNAs using the online tool CRISPR-Cas9 Guide RNA Design Checker provided by the Independent DNA Technologies (IDT) (https://www.idtdna.com/site/order/designtool/index/CRISPR_SEQUENCE). We pasted 0.5–1 kb DNA regions where we sought to identify gRNA binding sites and selected 20-bp sequences immediately preceding the protospacer-adjacent motif (PAM) for *Streptococcus pyogenes* Cas9 (5′-NGG-3′) without repetitive elements (e.g., Alu elements) or high-frequency single nucleotide polymorphisms (based on dbSNP13 entries). Binding sites were selected prioritizing on-target over off-target scores. The selected binding sites were the following: H2-018, TTTAACAAAATACTGGGAGG; H2-201, TGTATAAACATAAGGACTCT; H2-412, AGTCTTAACCCAAGGACTCC; H2-610, ATTCCTAGAGATTGTTCAAT; and H2-821, TTTACAATAAAGAGATGAAG.

To assemble RNPs, catalytically inactive *S. pyogenes* Cas9 (dCas9) was first expressed and purified (protocol provided upon request, but there are also alternative commercial options such as for example from New England Biolabs®). The crRNA and the tracrRNA with the Alt-R® modifications were purchased from IDT. To ensemble RNP complexes, crRNA and the tracrRNA for each site were first pre-mixed (200 nM final in each) in the IDT Duplex Buffer, heated to 95 °C for 5 min, and allowed to cool down to room temperature; then, crRNA/tracrRNA were mixed with dCas9 at 1:1 molar ratio (~ 200 nM) and incubated at room temperature for 15 min. RNPs should be used freshly assembled for best results. RNPs for all binding sites were combined and mixed with 1 μg of HMW genomic DNA (1 nM final in each RNP) in 50 μL CutSmart Buffer from New England BioLabs (Cat. #B7204) and incubated at 37 °C for 15 min. Genomic DNA complexed with RNPs was then digested with 40 U of Exonuclease I (Cat. #X8010; Enzymatics®), 100 U of Exonuclease III (Cat. #M0206L; New England Biolabs), and 20 U of Lambda exonuclease (Cat. #M0262S; New England Biolabs) simultaneously at 37 °C for 9 h in CutSmart Buffer. Exonucleases were inactivated at 80 °C for 20 min. Finally, digested DNA was purified by the magnetic SPRI method (0.5 × beads volume) and eluted in 25 μL 0.1 × TE buffer. We used 15 μL for TELL-Seq library preparation.

### Target amplification using long-range PCR

We followed PCR conditions previously established to amplify the 13 kb *SCN10A* amplicon and used the same conditions to also amplify three non-overlapping internal shorter regions: 3.9 kb, 4.1 kb, and 2.7 kb^43^. We failed to obtain PCR product from a fourth region that would have completed the full 13 kb region in shorter amplicons, but it was ultimately not necessary for our validation purposes. PCR reactions were conducted using NA12878 genomic DNA as template. For 13 kb *SCN10A* PCR we used Supreme NZYLong DNA Polymerase (Cat. #MB331, NZYTech®) according to the manufacturer’s recommendations. Cycling protocol: 94 °C for 5 min; 30 cycles of 94 °C for 20 s, 68 °C for 30 s, and 68 °C for 14 min; final step at 68 °C for 21 min. Shorter *SCN10A* amplicons were generated with Phusion Polymerase (Cat. #F565L, ThermoFisher Scientific®) according to the manufacturer’s recommendations. Cycling protocol: 98 °C for 30 s; 30 cycles of 98 °C for 8 s and 72 °C for 90 s; final step at 72 °C for 8 min. All PCR products were processed with ExoSap-IT (Cat. #78200.200.UL; ThermoFisher Scientific) to remove primers plus one or two rounds of 0.41 × HighPrep™ PCR Clean-up beads (Cat. #AC-60050; MagBio Genomics®) for 13 kb *SCN10A* amplicons. We used modified HighPrep™ PCR Clean-up beads procedures to remove large DNA molecules detected in the gel wells with shorter *SCN10A* amplicons, as shown in Fig. 5B. Specifically, the samples were diluted to 100 μL and 41 μL of beads were next added. After a 5 min incubation at room temperature, the tubes were placed on a magnetic stand. After a minute, the 141 μL of supernatant were pipetted into a new tube, and additional 60 μL of beads were added to it before proceeding with washes and elution as specified by the manufacturer. Although there was enough PCR product to proceed to library preparation, we warn that our specific clean-up procedure led to a high product loss; thus, we recommend trying alternative bead ratios for a better recovery.

Likewise, we followed PCR conditions previously established to amplify the 3.4 kb *PIK3CA* amplicon and used the same conditions to amplify the 1.8 kb *PIK3CA* amplicon^45^. HCC202 cells were used as cDNA source for the generation of *PIK3CA* amplicons. RNA was extracted using the *Quick*-RNA Miniprep Kit™ (Cat. #R1054; Zymo Research®) following the manufacturer’s recommendations. For cDNA generation, 1.5ug of RNA was processed using the SuperScript™ III First-Strand Synthesis System (Cat. #18080051; ThermoFisher Scientific) and either 1 µL of random hexamers or 1 µL of oligodT primer, as indicated in the Results section. PCR reactions were conducted using Phusion Hot Start II High-Fidelity polymerase (Cat. #F-565L; Thermo-Fisher Scientific) and 1 µL of stock or 1:20 dilution cDNA, as indicated in Fig. 5B. Cycling protocol: 98 °C for 30 s; 30 cycles of 98 °C for 10 s, 65 °C for 20 s, and 72 °C for 1 min; final step at 72 °C for 8 min. PCR products were cleaned up with ExoSap-IT (Cat. #78200.200.UL; Thermo-Fisher Scientific).

All PCR products were assessed by gel electrophoresis and quantified using the Qubit™ dsDNA HS Assay kit (Cat. #Q32854; Thermo-Fisher Scientific). Primers were synthesized by Integrated DNA Technologies® and diluted to 10uM in 0.1 × TE buffer. Primer sequences (5′–> 3′): 13 kb *SCN10A* (forward) GCCATGACCATTGTTATTTGTCCAGA and (reverse) CCTGAAGAAATGTCACGGCTTGTTAG^43^; 3.9 kb *SCN10A* (forward) CACTTTGCACGAAGTGCTTG and (reverse) GCCCACACACCTCTCTTCAT; 4.1 kb *SCN10A* (forward) GTGTGGGCTCTTGCTCTCAT and (reverse) GAGGTGGGAGGATGACTTGA; 2.7 kb *SCN10A* (forward) TGTAATTTCTGCAGCCACGA and (reverse) CACTGGTTTCCCATTGCTCT; 3.4 kb (cDNA) *PIK3CA* (forward) TGGGACCCGATGCGGTTA and (reverse) AATCGGTCTTTGCCTGCTGA^45^; 1.8 kb (cDNA) *PIK3CA* (forward) CAGACGCATTTCCACAGCTA and (reverse) TGTGACGATCTCCAATTCCCA.

### Targeted TELL-seq protocol with Cas9-based enriched (impure) targets

Linked-read libraries were generated with an adapted version of the WGS TELL-seq Library Prep kit described here (Standard Bundle, Cat# 100035, 100036, 100003, 100004; Universal Sequencing Technology Corp.) following the Library Prep User Guide (version 8.0) and the amounts and volumes validated here for targeted phasing: 100 pg of HLS-CATCH-enriched single targets, 270 pf of a five-target HLS-CATCH-enriched panel or 15 μL of CaBagE-enriched four-target panel; 6 μL of TELL microbeads; 25 μL barcoding reaction at 35 °C for 15 min followed by stabilization at 35 °C for 30 min and the tagging/exonuclease reaction 35 °C for 10 min. Following the wash steps as described according to the manufacture’s protocols with 13 amplification cycles (for the target panel) in 25 μL reactions. The PCR products were purified twice by the magnetic SPRI method (0.78 × beads volume) and eluted in 25 μL low TE. The average size of the library was determined to be 447 bps by the TapeStation D1000 ScreenTape system (Agilent®), and the concentration was determined to be 2–4 nM by the Qubit High-sensitivity dsDNA Assay. Libraries (2 × 145 bp Illumina-compatible paired-end reads) were sequenced on a MiSeq® instrument (Illumina) using the MiSeq reagent Micro kit v2, 300 Cycles (Cat. #MS-103–1002; Illumina).

### Targeted TELL-seq protocol with PCR products

Linked-read libraries were generated with an adapted version of the WGS TELL-seq Library Prep kit described here (Standard Bundle, Cat# 100035, 100036, 100003, 100004, Universal Sequencing Technology Corp.) following the Library Prep User Guide (version 8.0) and the amounts and volumes validated here for amplicon phasing (below). Bacterial and viral genomic (filling) DNAs were used to minimize human DNA collisions. The *Escherichia coli* DH10B (bacterial) strain was purchased from New England Biolabs (Cat. #FEREC0113), and genomic DNA was extracted using the *Quick*-DNA Miniprep Kit (Cat. #D3024; Zymo Research) following the manufacturer’s recommendations. BstI P-digested lambda phage gDNA was purchased from TaKaRa® (Cat. #3402).

Amplicon TELL-Seq libraries were generated combining 20 pg of a single PCR product, 60 pg *E.coli* genomic DNA or 80 pg BstP I-digested lambda phage genomic DNA, and 6 μL of TELL microbeads (3 million microbeads), as indicated. When testing a target panel, we combined equal amounts of each fragment (for a total of 80 pg) and 6 μL of TELL microbeads (without adding filling DNA. In all tests, we used approximately 75,000 TELL microbeads for indexing (taking 1/40th of the processed bead solution; and libraries were amplified with 18–21 PCR cycles.

### Sequencing

Libraries were sequenced on a MiSeq instrument (Illumina) using the MiSeq reagent Micro kit v2, 300 Cycles (Cat. #MS-103-1002; Illumina), 2 × 145 bp Illumina-compatible paired-end reads. Index 1 (I1) contains 18-base TELL bead sequences (linked-read barcodes). Index 2 (I2) contains 8-base sample indexes for multiplexing. Reads R1 and R2 contain paired-end DNA inserts.

### Linked-read data analysis

Sequencing data was processed using the TELL-Seq v1.1.1 software, which is available for free from the link universalsequencing.com/pages/tell-seq-software (Supplementary Fig. S7). This software integrates third-party and open-source tools that, combined, enable the processing of linked-read sequencing data starting with sequencing images and ending with haplotype phasing and visualization on IGV. Briefly, TELL-Seq v1.1.1 uses Illumina’s bcl2fastq-v1.8.4 for BCL-to-FASTQ file conversion and sample demultiplexing. It also uses FastQC-v0.12.0 for sequencing quality control (developed by github/s-andrews) and cutadapt-v2.5 for adapter trimming (developed by github/marcelm). For cutadapt, we used—a option for 3′ adapter trimming and − O5 for a minimum overlap threshold of 5 nucleotides. Taken into account for the possibility of adapter dimers, three different sequences were used for R1 trimming (TGAAGCGGCGCACGAAAAACGCGAAAGCGTTTCAC, ATCACGGACTGCCCATAGAGAGGCTCTGG, and TGGTCATGTGGAGACGCTGGG), and one sequence was used for R2 trimming (TGGGCCGGTGCAGTTAATGTAGGGAAAGAGTGT). TELL-Seq v1.1.1 has also a step of TELL-bead barcode correction for single-read barcodes with one nucleotide difference with a multi-read barcode. This correction assumes that the nucleotide difference is likely a sequencing error. TELL-Seq v1.1.1 uses BWA-MEM-v0.7.17-r1188 for read alignment. Reads are mapped against the reference human genome (hg38) using the following options: − B 4 for mismatch penalty, − O 6 for gap open penalty, − E 1 for gap extension penalty, and − A 1 for matching score. More detailed information about mapping options can be found in our previous work (Ref.^24^ and in User Guides that can be frely downloaded from Universal Sequencing Technology’s website: universalsequencing.com/pages/tell-seq-software). For duplicate identification within barcodes and removal, as well as diploid variant calling, TELL-Seq v1.1.1 uses Picard (MarkDuplicates) and HaplotypeCaller, both from The Genome Analysis Toolkit (GATK)-v3.8-1-0-gf15c1c3ef. With MarkDuplicates, only duplicates within a barcode were identified. With HaplotypeCaller, mapped reads are processed as diploid using the –sample-ploidy − 2 option and the rest of default options. Lastly, TELL-Seq v1.1.1 uses HapCUT2-v1.3 for phasing and estimation of haplotype blocks. HapCUT2 required two extra steps. On step to convert BAM file to a compact fragment file format containing only haplotype-relevant information (./build.extractHAIRS –10 × 1 –bam reads.sorted.bam –VCF variants.VCF –out unlinked_fragment_file). The second step uses the command LinkFragments to link reads (python3 utilities/LinkFragments.py –bam reads.sorted.bam –VCF variants.VCF –fragments unlinked_fragment_file –out linked_fragment_file). After removal of homozygous sites and these two steps, the linked-read fragments and variants in VCF format become input files for HapCUT2, which we used following HapCUT2 instructions (e.g., using 6.98 as default SNV pruning setting)^55^. For haplotype block assembly, we used 40 kb as distance (d), which should not negatively affect the analysis of shorter targets (.build/HAPCUT2 –nf 1 –fragments linked_fragment_file –VCF variantcalls.vcf –output haplotype_output_file_path -d 40000). We did not consider short indels, focusing on SNVs only (–indels 0) and using –tags 1 and –maxfragments 10000000 options. Allele frequency threshold was set to 0.1. We note that we used the target sequence as reference (not the whole human genome) for cDNA-amplified *PIK3CA* targets, and we used the fourteen non-overlapping fragments resulting from BstP I digestion for lambda phage DNA samples. Lastly, we also used 10 × Genomics LongRanger v2.2.2 for the analysis of whole-genome TELL-Seq data after data conversion into 10X Genomics format and using the GRCh38-2.1.0 draft as reference.

We ran the following code to run TELL-Seq v1.1.1, which can also be used to replicate the results (Note: information about software installation can be found in the link universalsequencing.com/pages/tell-seq-software):
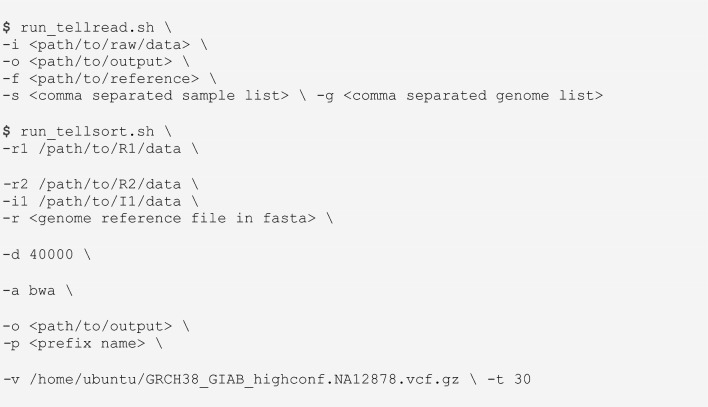


### TELL-Seq data visualization

The Integrative Genomics Viewer, IGV-v2.11.1 (Broad Institute) was used for data visualization assisted with WhatsHap-v1.1 (github/whatshap) and it is now part of the TELL-Seq v1.1.1 software. The TELL-Seq software generates output files that can be uploaded as tracks on IGV. These tracks display read mapping, phased alleles, and targeted haplotypes. For Supplementary Fig. S1C, visualization was done using 10 × Genomics Loupe Browser, which displays gene annotations and linked-read density heatmaps.

### Analysis of site behaviors for Cas9-enriched targets

We processed Cas9-enriched targets as follows. Several pieces of customized scripts have been developed so that TELL-Seq reads can be aligned to the targeted regions in the reference genome for site behavior extractions from read aligned bam and phased variant vcf files. Briefly, read-aligned bam with duplicates removed from the Tell-Sort analysis were filtered using samtools “view” program so that only the reads aligned to the targeted regions were kept. The CIGAR string from each read in this filtered bam were parsed so that where each nucleotide in the read been mapped to the reference was determined. With this process, the coverage with different nucleotides on each position in the targeted region could be counted. SNVs were detected through the Tell-Sort analysis, either phased or not phased, in the vcf format, were also mapped to the sites in the targeted regions. As a standard of the comparison, this analysis was also applied to bam file with the reads from 10 × Genomics, which is downloaded from https://github.com/genome-in-a-bottle/giab_data_indexes/blob/master/AshkenazimTrio/alignment.index.AJtrio_10Xgenomics_ChromiumGenome_GRCh37_GRCh38_06202016.HG002 (GRCh38) combined with the phased vcf file from GIAB, which is downloaded from https://ftp-trace.ncbi.nlm.nih.gov/giab/ftp/release/AshkenazimTrio/HG002_NA24385_son/latest/GRCh38/SupplementaryFiles/(HG002_GRCh38_1_22_v4.2.1_benchmark_hifiasm_v11_phasetransfer.vcf.gz).

### Supplementary Information

Supplementary Figures.

## Data Availability

Datasets will be made available at GEO NCBI under Accession Number PRJNA771708.
