# Supplementary material for: Targeted phasing of 2–200 kilobase DNA fragments with a short-read sequencer and a single-tube linked-read library method
Source: Sci Rep. 2024 Apr 5;14:7988. doi: 10.1038/s41598-024-58733-0 (PMC10997766; doi:10.1038/s41598-024-58733-0)
Supplement: Supplementary file 1 — Supplementary Figures. [file 41598_2024_58733_MOESM1_ESM.docx]

**Targeted Phasing of 2-200 Kilobase DNA Fragments with a Short-Read Sequencer and a Single-Tube Linked-Read Library Method**

**Veronika Mikhaylova^1,*^, Madison Rzepka^1,*^, Tetsuya Kawamura^1,*^, Yu Xia^1,*^, Peter L. Chang^1^,**

**Shiguo Zhou^2^, Amber Paasch^1^, Long Pham^1^, Naisarg Modi^1^, Likun Yao^3^, Adrian Perez-Agustin^4^,**

**Sara Pagans^4^, T. Christian Boles^2^, Ming Lei^5^, Yong Wang^5^, Ivan Garcia-Bassets^1,#^, and Zhoutao Chen^1,#^**

**SUPPLEMENTARY FIGURES**

**FIGURE S1**


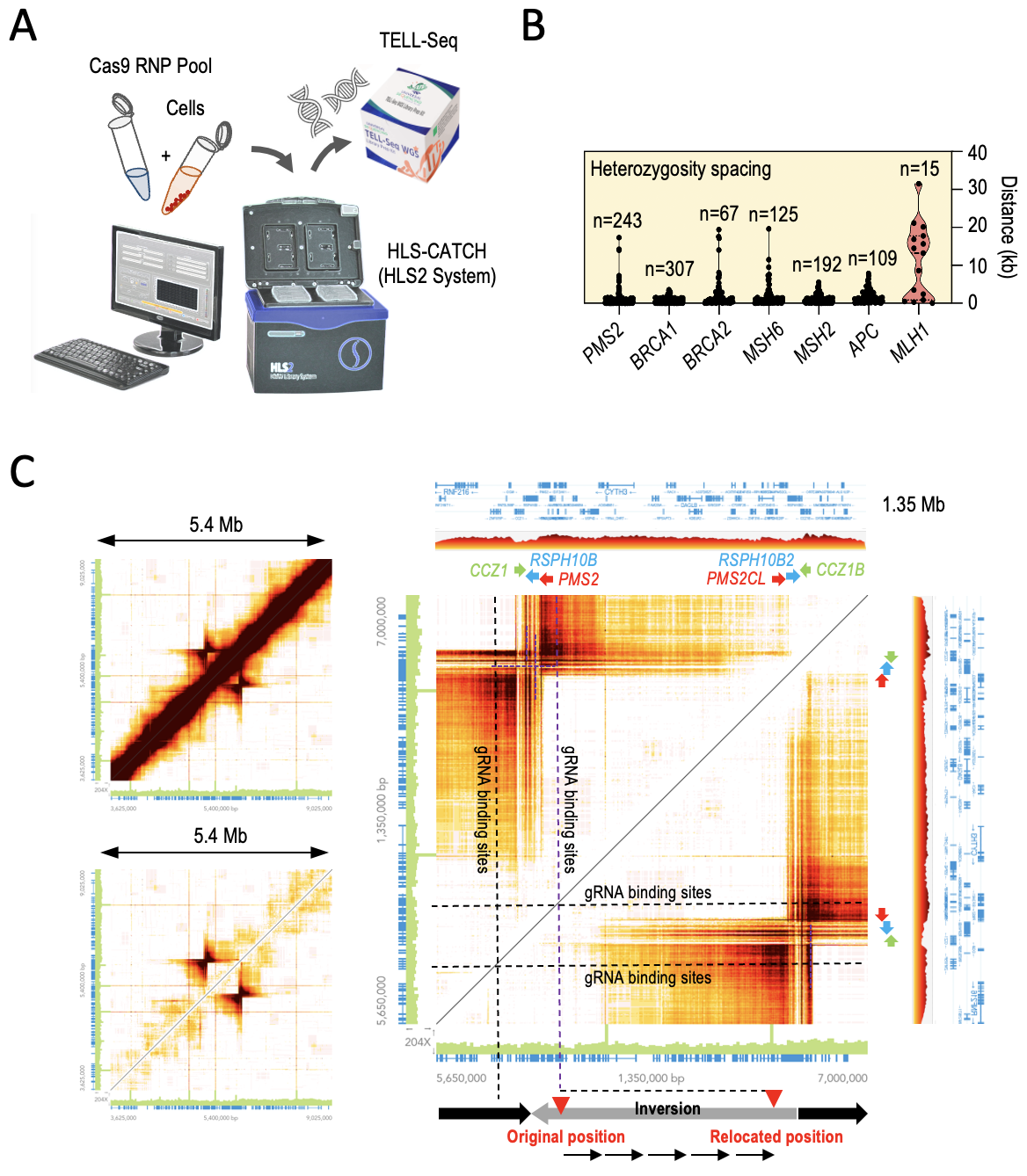


Singularities in the *MLH1* and *PMS2* loci in HG002. (A) HLS-CATCH system (Sage Science). (B) Violin plots showing spacing between adjacent GIAB-annotated heterozygous sites across the seven targets: *MLH1*, n=15 (datapoints), *APC*, n= 109; *MSH2*, n=192; *MSH6*, n=125; *BRCA2*, n=67; *BRCA1*, n=307; *PMS2*, n=243. (C) WGS 10X Genomics linked-read data visualized with LongRanger Loupe (10X Genomics) focusing on the *PMS2* locus. Biding sites for gRNAs in the HLS-CATCH system are indicated. Data source: Human Pangenome Reference Consortium (<https://github.com/human-pangenomics/HG002_Data_Freeze_v1.0>). (*Small heatmaps*) GRCh38 chr7:3,625,000-9,025,000. Local signal (diagonal) masked in the bottom heatmap for better visualization. (*Large heatmap*) Genomic coordinates, GRCh38 chr7:5,650,000-7,000,000. Scheme of the inversion shown at the bottom. Signs of the inversion that relocates the 3’ gRNA sites ~700 bp away (red arrowheads) from its position in the reference genome (purple dashed vertical line). The inversion, recurrent in the human population according to Ref. 31, is flanked by long segmental duplications consisting in the *CCZ1*, *RSPH10B*, and *PMS2* genes and the *PMS2CL* pseudogene and the *RSPH10B2* and *CCZ1B* genes with inverted orientation in the reference genome.

**FIGURE S2**


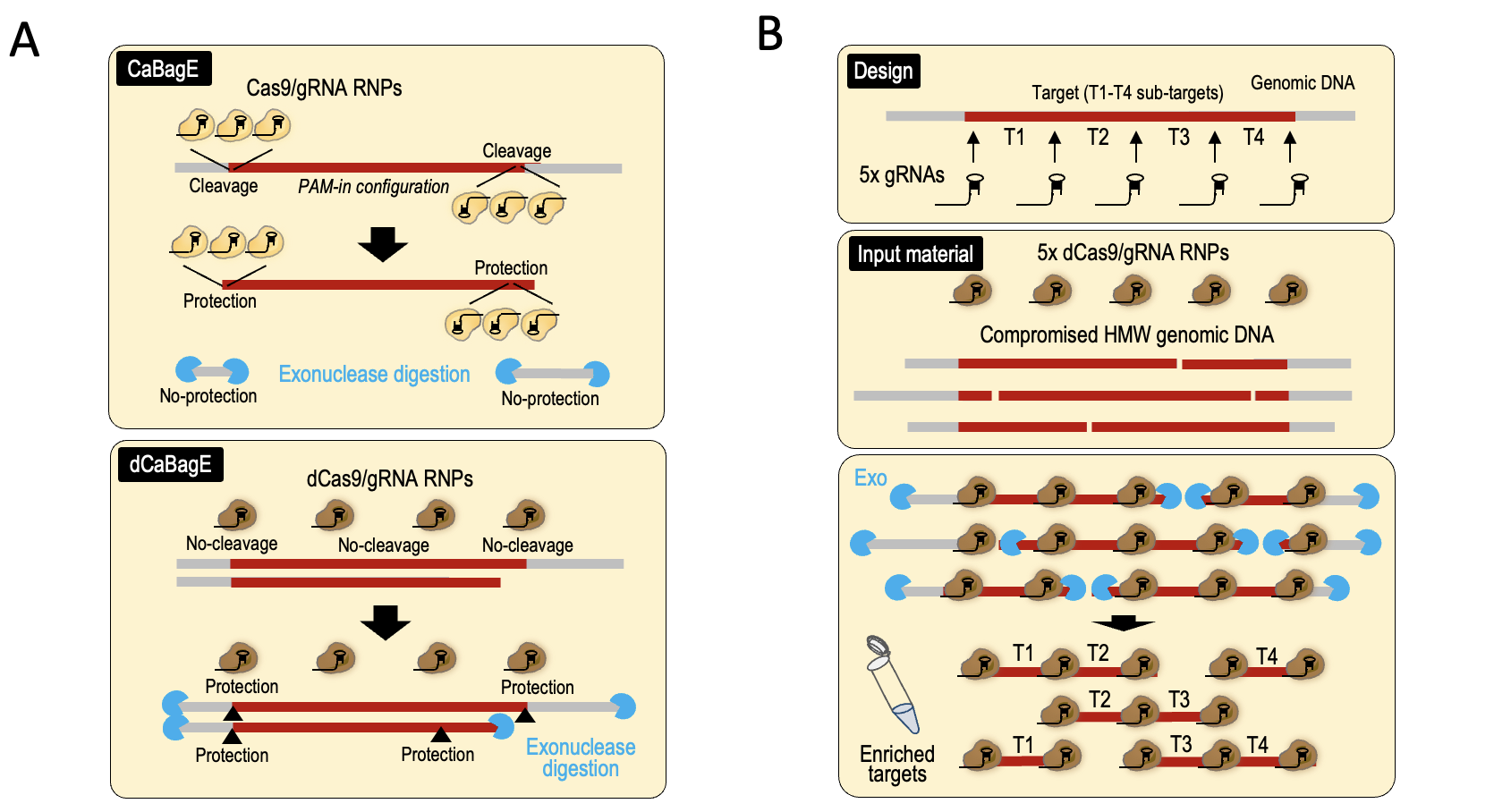


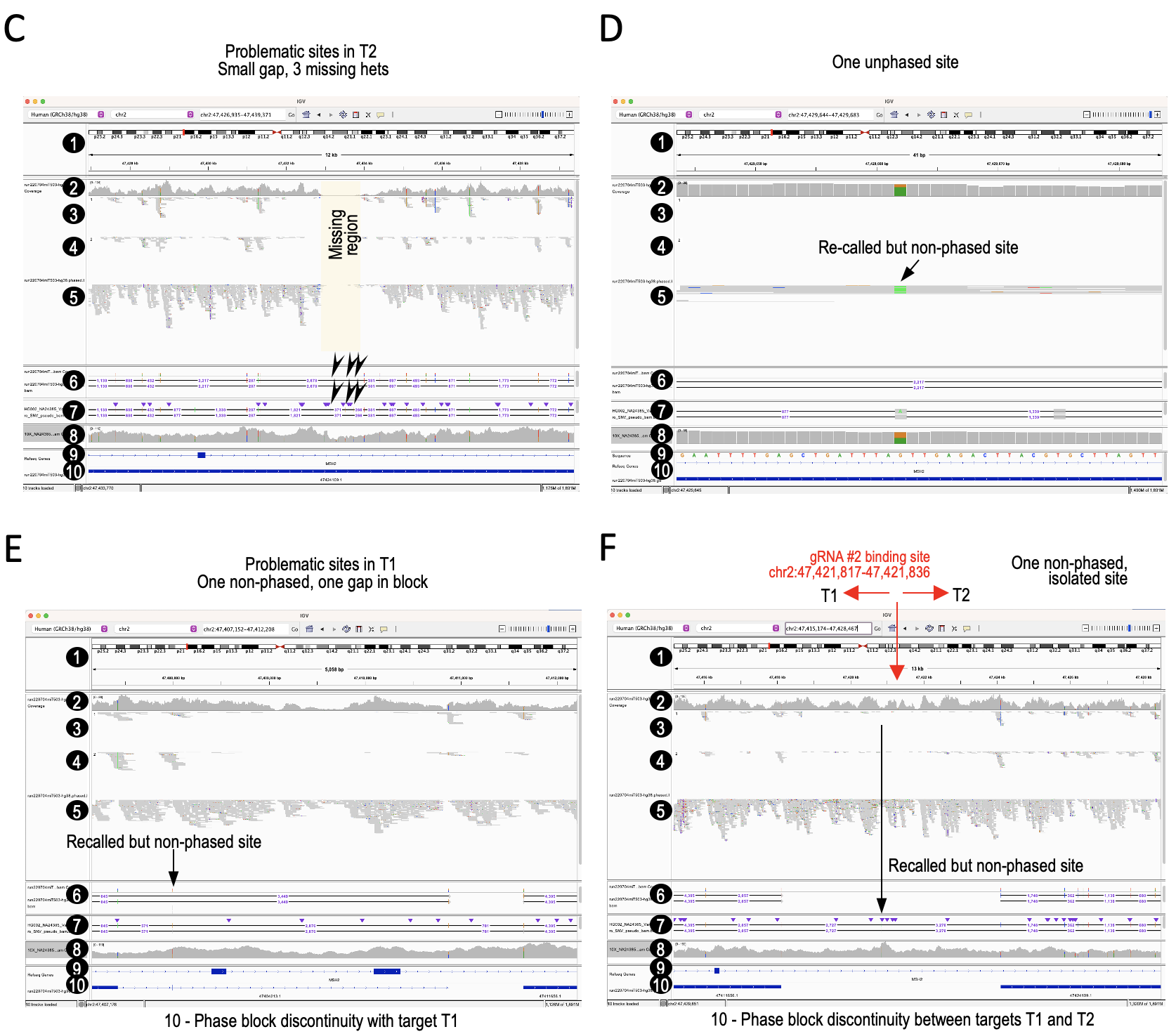


CaBagE and dCaBagE strategies and problematic sites along the T2 and T1 sub-targets. (A) With CaBagE, multiple Cas9 molecules are guided to the two ends of the target in PAM-in orientation for best results according to Ref. 34. After Cas9-mediated cleavage, DNA-bound Cas9 molecules protect the target from subsequent exonuclease digestion, leading to target enrichment over non-targeted DNA. In cases when the integrity of the target might be compromised, targeting the two ends may result in low target recovery. With dCaBagE, dCas9 is guided internally in addition to the ends of the target. DNA-bound dCas9 molecules can then protect the full target but also shorter target fragments from subsequent exonuclease digestion. (C-F) Screenshots from IGV portal showing TELL-Seq data from the four *MSH2* sub-targets (T1-T4). Tracks are labeled on the first screenshot for all screenshots. Phase block shown at the bottom as a blue bar. (C, D) In T2, three heterozygous sites (pointed by arrows) were missed, explained by the presence of a small gap (no read coverage) in the HG002 cells used in this study (in C), and one heterozygous site was correctly re-called by not phased (shows as part of the bulk of unphased reads) (in D). (E, F) In T1, one site was correctly re-called but not phased and there was a discontinuity in the phase block likely as the two underlying sites were not phased together (in E). Another correctly re-called heterozygous site was not phased, located at the end of the target and relatively isolated (in F). Common labeling in C-F (tracks): 1- Genomic coordinates; 2 - Sequencing coverage; 3 - Phased reads in haplotype 1; 4 - Phased reads in haplotype 2; 5 - Unphased reads; 6 - Haplotype annotations according to TELL-Seq experiment; 7 - Haplotype annotations according to GIAB; 8 - Sequencing coverage in GIAB data; 9 - Gene (intronic and exonic) annotations; 10 - Phase blocks (one single line represents a single block)

**FIGURE S3**


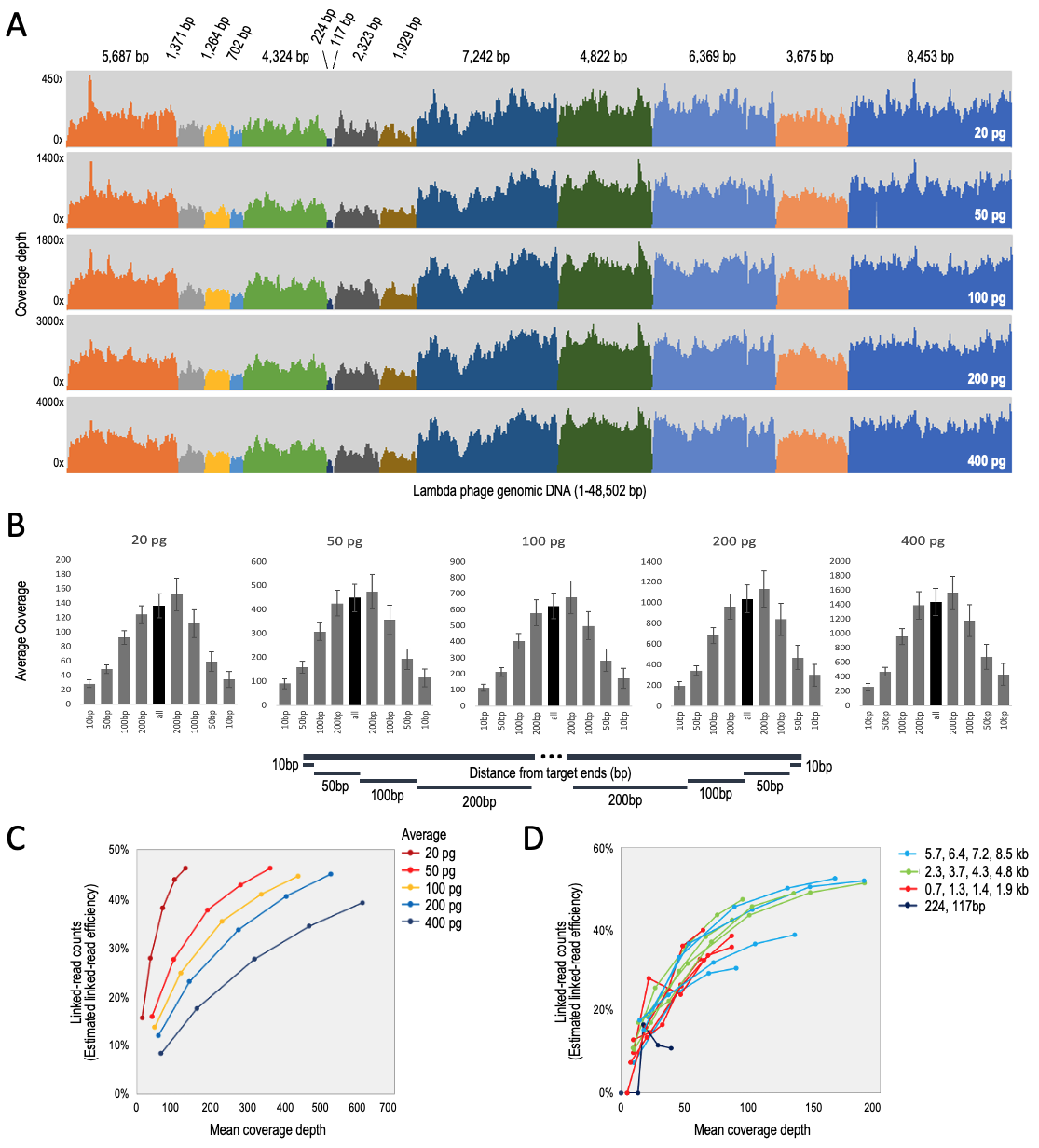


TELL-Seq analysis of BstP I-digested lambda phage genome. (A) Coverage across fragments at nucleotide resolution. Fragment sizes indicated on top. Input amounts indicated on every track. (B) Average coverage in selected regions by input amounts: 5’ terminal 10 bp, 5’ 11bp to 50 bp, 5’ terminal 51 bp to 100 bp, 5’ terminal 101 to 200 bp, rest of the fragment, 3’ terminal 101 to 200 bp, 3’ terminal 100 bp to 51 bp, 3’ 50 bp to 11 bp, 3’ terminal 10 bp. Scheme shown at the bottom. Data represents average of all 13 fragments (excluding 117 bp). Error bars represent S.E.M. (C, D) Linked-read efficiencies by input after subsampling for the average from all fragments (as a %) in C. Subsampling (100%, 75%, 50%, 25%, 10%). And linked-read efficiencies by fragment size in the 20 pg sample.

**FIGURE S4**


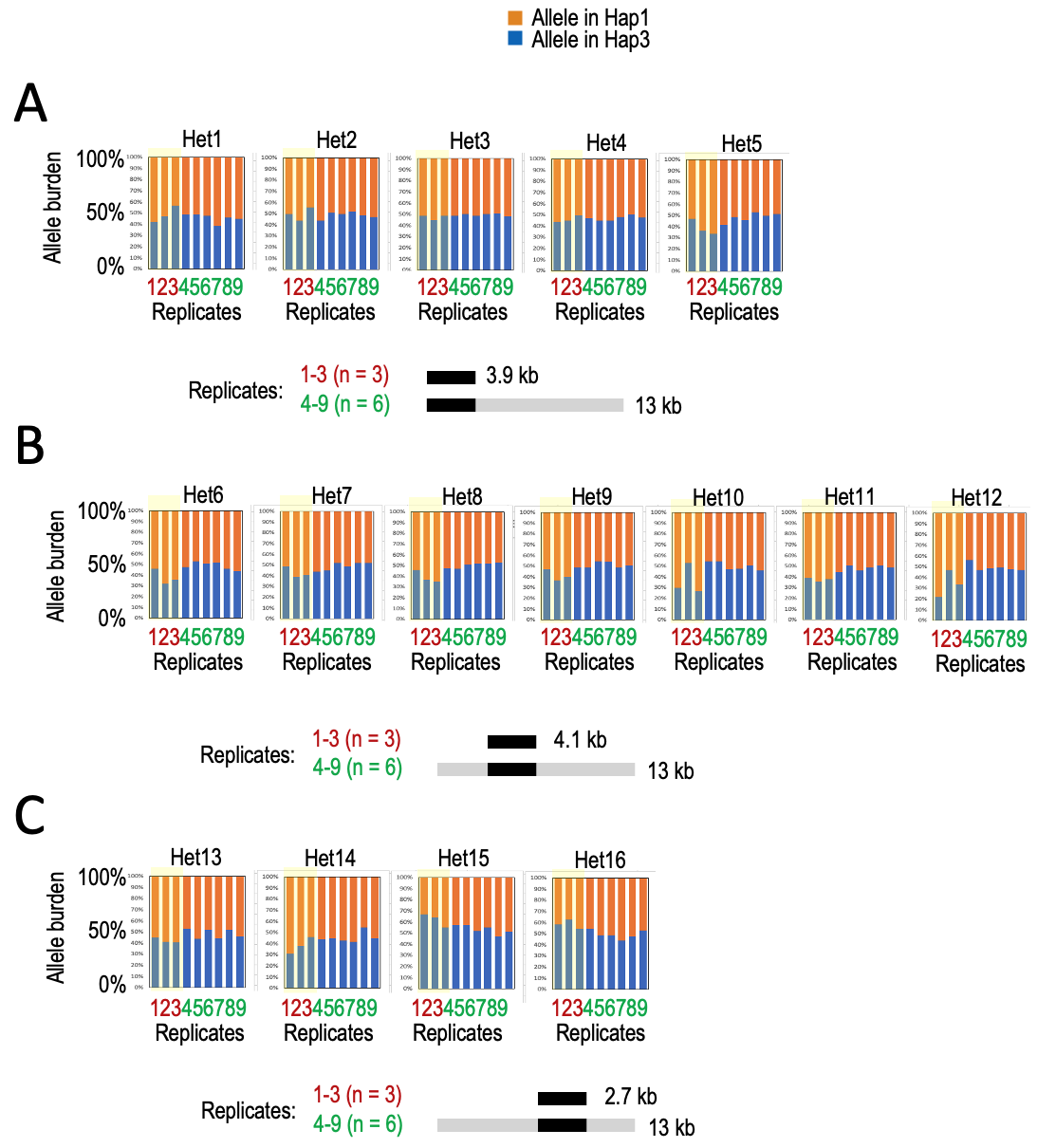


Amplicon phasing. (A-C) Allele burden for 16 heterozygous sites in HG001 comparing multiple PCR products (three replicates for short *SCN10A* amplicons and six replicates for 13 kb *SCN10A* amplicons, as indicated on the right). In general, allele burden fluctuates around 50%, indicating a balanced amplification in which maternal and paternal haplotypes are similarly amplified (Hap1 vs. Hap3). Columns 1-3 represent replicates of the short amplicon (n=3). Columns 4-9 represent replicates of the full 13 kb fragment, three replicates using lambda phage genome as filling DNA (4-6) and 13 kb fragment *E. coli* genome as filling DNA (7-9). Heterozygous sites are organized by their position along the target, see schemes (A-C).

**FIGURE S5**


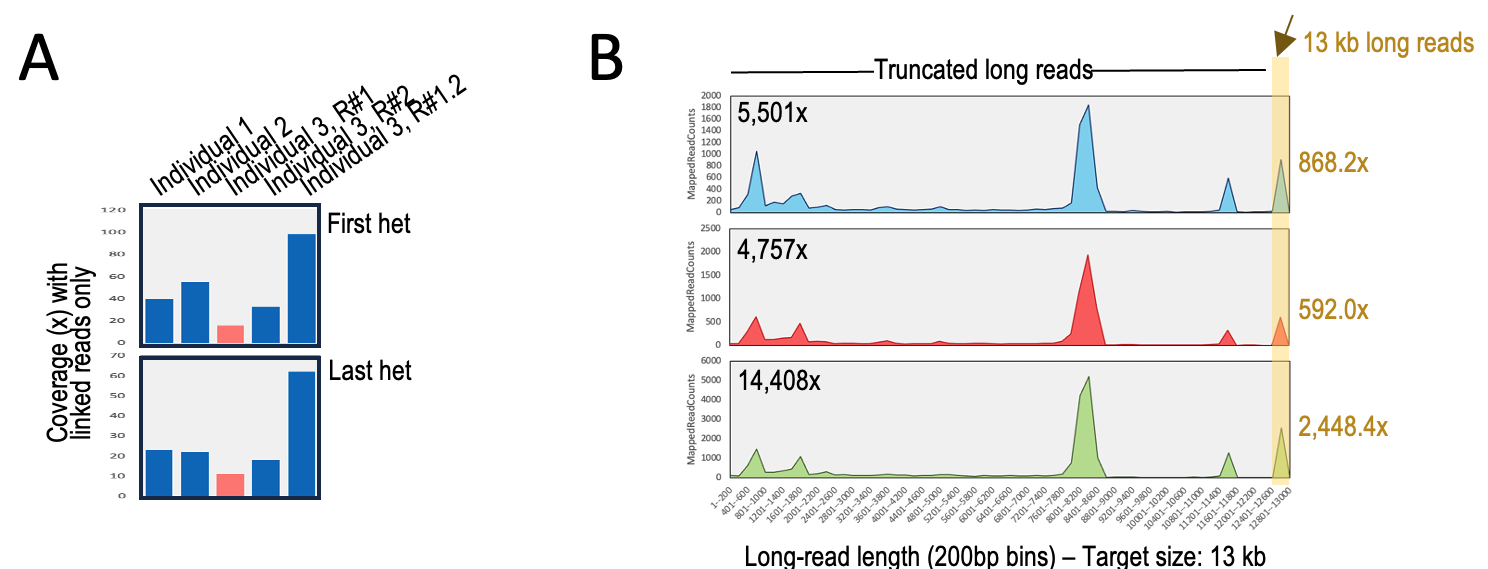


Targeted TELL-Seq of peripheral blood-extracted genomic DNA from Hap1/Hap3 carrier individuals. (A) Linked-read coverage in the first and last heterozygous sites in the libraries shown in A. The sites with phasing issues correspond to the sites with the lowest linked-read coverage. (B) Long-read counts (200 bp bins) in ONT data generated by Ref. 43. Highlighted coverage from 13 kb long reads.

**FIGURE S6**

Impact of sequencing depth on phasing and genotyping accuracies. Data based on *SCN10A* amplicons (n= 6 libraries with 13 kb and n = 3 libraries with smaller fragments). Genotyping and phasing qualities were compared for all the libraries using 100% of the reads and after subsampling each library at 50%, 25%, 12.5%, 6.25%, 3.125%, 1.56%, and 0.78%. In total, this plot compares n= 72 ‘experiments’ (n = 9 libraries x 8 full and subsampled conditions). Subsampling reads is expected to negatively impact the genotyping and phasing performances. We aimed to determine the sequencing depth at which genotyping and phasing errors emerge. Violin plots containing box plots show on-target read counts (left axis) and coverage (right axis) organized by genotyping and phasing qualities (groups 1-4). Group 1 (as also indicated on the right) represents situations with correct genotyping and phasing (i.e., all heterozygous sites are correctly recalled without phasing errors). Group 2 represents situations with correct genotyping but phasing errors—switch errors (i.e., all heterozygous sites are correctly recalled but some are incorrectly phased). Group 3 represents situations with some genotyping errors (i.e., some heterozygous sites have been missed, which makes phasing not possible on these sites). Group 4 represents situation with many genotyping errors.


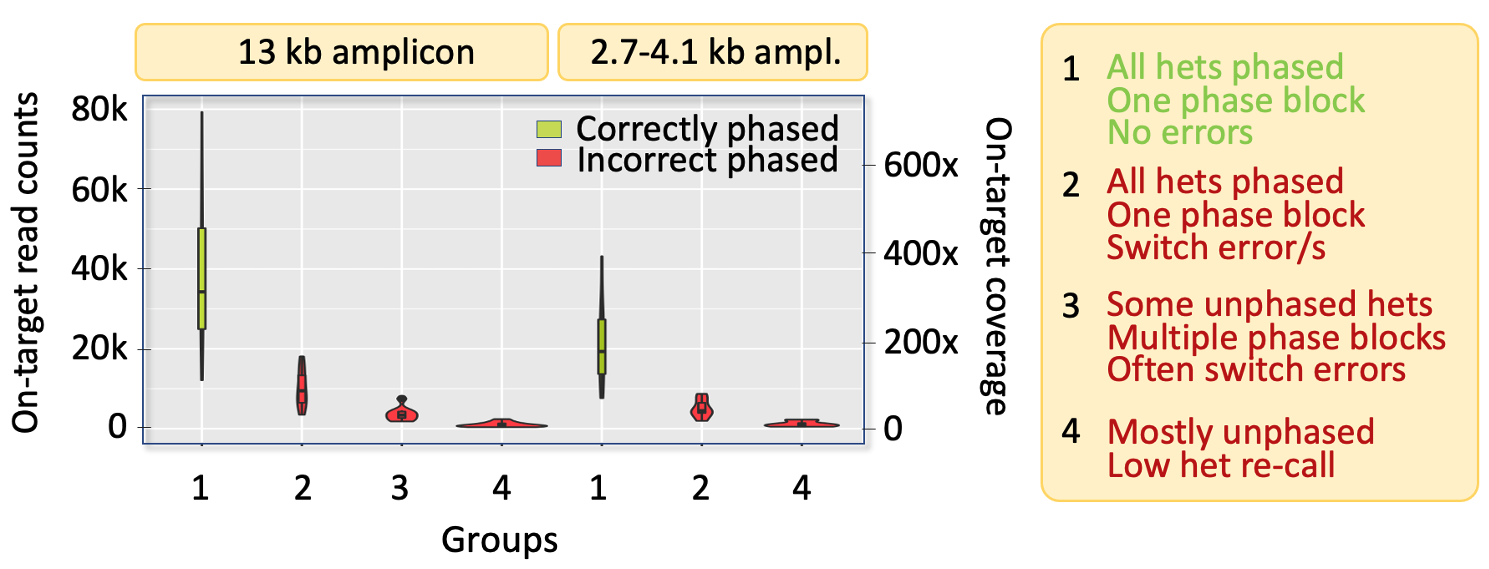


**FIGURE S7**


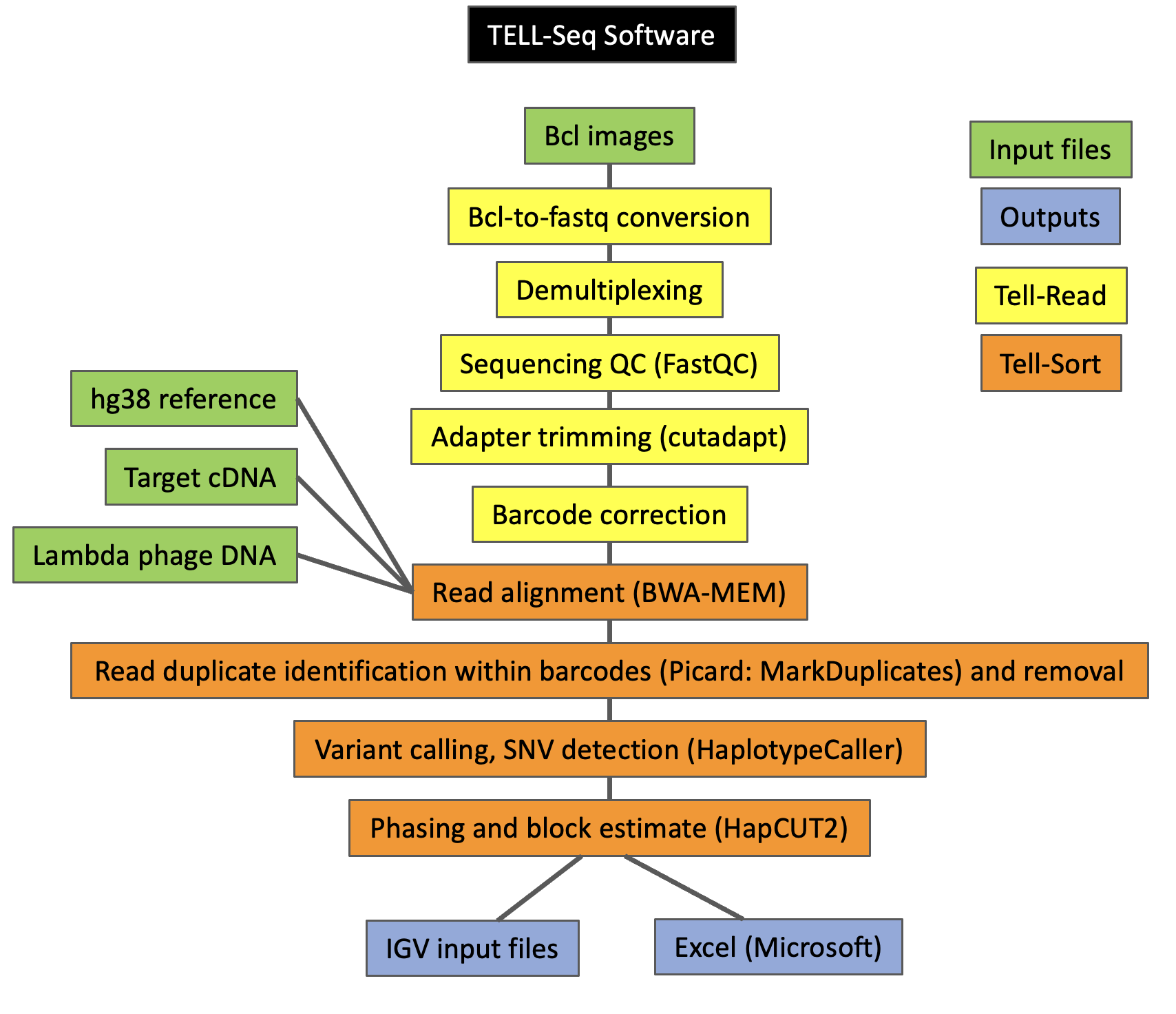


Workflow depicting the phasing process (see Methods). The TELL-Seq software can be divided into two distinct functional modules: Tell-Read for the generation of fastq files and read preparation for mapping, and Tell-Sort for mapping and phasing.
